# Non-canonical activation of MAPK signaling by the lncRNA *ASH1L-AS1*-encoded microprotein APPLE through inhibition of PP1/PP2A-mediated ERK1/2 dephosphorylation in hepatocellular carcinoma

**DOI:** 10.1186/s13046-025-03465-w

**Published:** 2025-07-11

**Authors:** Lei Zhao, Ke Si, Shenjian Luo, Lantian Zhang, Shuai Mao, Wenliang Zhang

**Affiliations:** 1 The Key Laboratory of Advanced Interdisciplinary Studies, The First Affiliated Hospital of Guangzhou Medical University, GMU-GIBH Joint School of Life Sciences, The Guangdong-Hong Kong-Macao Joint Laboratory for Cell Fate Regulation and Diseases, Guangzhou Medical University, Guangzhou, 510182 People’s Republic of China; 2 Department of Endocrinology and Metabolism, Nanfang Hospital, Southern Medical University, Guangzhou, 510515 People’s Republic of China; 3 Department of Clinical Oncology, The University of Hong Kong-Shenzhen Hospital, Shenzhen, 518053 People’s Republic of China; 4 Department of Hepatic Surgery, the First Affiliated Hospital, Sun Yat-sen University, Guangzhou, 510080 People’s Republic of China

**Keywords:** *ASH1L-AS1*, Liver cancer, LncRNA encoded-microprotein, MAPK signaling, PP1/PP2A

## Abstract

**Background:**

MAPK/ERK1/2 signaling is often activated in hepatocellular carcinoma (HCC), yet classical RAS-RAF-MEK mutations are rare, indicating the involvement of non-canonical regulatory mechanisms. Long non-coding RNAs (lncRNAs) can encode microproteins that play key roles in cancer. LncRNA *ASH1L-AS1* has coding potential, but its role in HCC remains unclear. Clarifying its role in MAPK signaling may uncover novel therapeutic targets for HCC.

**Methods:**

Translatable lncRNAs associated with HCC were identified by integrating data from the TCGA-LIHC cohort and the TransLnc database. The functional role of *ASH1L-AS1* and its encoded microprotein APPLE was explored through in vitro and in vivo assays, such as CCK-8, EdU incorporation, wound healing, Transwell migration and invasion, and xenograft tumor models. Mechanistic investigations were conducted to elucidate molecular mechanisms and identify potential therapeutic strategies, including co-immunoprecipitation, mass spectrometry, ChIP-qPCR, luciferase reporter assays, truncation mutation analysis, immunofluorescence, Western blot, RNA sequencing, drug sensitivity analysis etc.

**Results:**

A total of 696 translatable lncRNAs associated with HCC were identified, with their encoded products exhibiting specific subcellular localization. Among them, *ASH1L-AS1* stood out due to strong translational evidence and its significant association with disease progression, poor prognosis, immunosuppressive tumor microenvironment, and estrogen signaling. We confirmed that *ASH1L-AS1* encodes a microprotein, APPLE, which is stably expressed in HCC cells and consistently upregulated in tumor tissues regardless of RAS mutation status. Functionally, APPLE promotes ERK1/2 phosphorylation, activates MAPK signaling, and enhances HCC cell proliferation, migration, invasion, and tumor growth—effects reversed by APPLE knockdown or ERK1/2 inhibition. Mechanistically, APPLE binds to ERK1/2 and phosphatases PP1/PP2A, preventing ERK1/2 dephosphorylation and sustaining MAPK pathway activation. Additionally, the transcription factor E2F1 directly binds to the *ASH1L-AS1* promoter (− 300 to − 290 bp), upregulating APPLE expression and further amplifying ERK1/2 signaling. Drug sensitivity analysis identified 220 treatment combinations potentially effective against HCC subtypes driven by hyperactivation of the E2F1–ASH1L-AS1/APPLE–ERK1/2 axis.

**Conclusions:**

This study characterized APPLE as a novel oncogenic microprotein encoded by lncRNA *ASH1L-AS1*, uncovering a non-canonical mechanism of MAPK activation in HCC. The identified E2F1–ASH1L-AS1/APPLE–ERK1/2 signaling axis provides new insights into HCC pathogenesis and represents a promising target for precision therapy, though further validation in clinical cohorts and preclinical studies is needed.

**Supplementary Information:**

The online version contains supplementary material available at 10.1186/s13046-025-03465-w.

## Background

Primary liver cancer remains a major global health burden with rising incidence and mortality rates [1, 2]. Hepatocellular carcinoma (HCC) accounts for approximately 90% of cases [3]. Despite progress in early diagnosis and treatment, the complexity of HCC and the limitations of targeted therapies continue to pose significant challenges. The asymptomatic nature of early-stage HCC and the poor efficacy of late-stage treatments contribute to its high mortality rate [1]. Unraveling the molecular mechanisms driving HCC and identifying novel therapeutic targets are crucial for advancing innovative treatment strategies.

Long non-coding RNAs (lncRNAs) are emerging as key regulators in cancers, including HCC [4, 5]. Although traditionally regarded as non-coding, lncRNAs have recently been found to harbor non-canonical open reading frames (ORFs) that encode functional microproteins or polypeptides [6–9]. These translational products are widely involved in key biological processes such as cell proliferation, apoptosis, migration, invasion, and immune regulation [7–11]. Although studies have explored lncRNA-encoded microproteins in HCC [10], their broader biological significance remains largely uncharacterized.

The conserved lncRNA *ASH1L-AS1* remains poorly characterized, with only two studies to date investigating its roles in other types of cancer. One study demonstrated that *ASH1L-AS1* encodes a 90-amino acid microprotein, APPLE, which regulates translation initiation and promotes hematologic malignancies [12]. Another study suggested that in gastric cancer, *ASH1L-AS1* functions as a lncRNA, interacting with the NME1 protein to indirectly regulate oncogenic RAS signaling [13]. These findings underscore the functional heterogeneity of *ASH1L-AS1* across different cancer types; however, its specific role in HCC remains unclear. To date, the expression patterns, regulatory mechanisms, and biological functions of *ASH1L-AS1* in HCC have not been thoroughly investigated. Several key questions remain open: Is *ASH1L-AS1* aberrantly expressed in HCC? What are the upstream regulators of its expression? And does it promote HCC progression by encoding the microprotein APPLE or through an RNA-mediated mechanism? A systematic exploration of these questions is essential to elucidate the role of *ASH1L-AS1* in HCC and may provide novel insights into disease pathogenesis as well as potential therapeutic avenues.

The Mitogen-Activated Protein Kinase (MAPK) signaling pathway is one of the most well-characterized in cancer biology, with its dysregulation contributing to over 40% of human cancers and being closely linked to various developmental disorders [14]. Extracellular signal-regulated kinases 1 and 2 (ERK1/2), encoded by *MAPK3* and *MAPK1*, are key components of the MAPK pathway, which is aberrantly activated in over 75% of HCC cases [15]. Although this pathway is conventionally driven by the Ras-Raf-MEK cascade, activating mutations in RAS and RAF—which are frequently observed in other cancers—are relatively rare in HCC [15, 16]. Furthermore, most inhibitors targeting this canonical axis have shown limited clinical efficacy in HCC [15], suggesting the involvement of alternative, non-canonical mechanisms in MAPK hyperactivation.

Recent studies have revealed that certain lncRNA-derived microproteins can influence the Ras-Raf-MEK cascade and promote MAPK activation, thereby facilitating tumor progression [17, 18]. However, it remains unclear whether these microproteins can regulate MAPK signaling through non-canonical pathways—particularly by directly modulating the activity of protein phosphatases involved in ERK1/2 dephosphorylation. Elucidating such non-canonical regulatory mechanisms is critical for advancing our understanding of HCC pathogenesis and may lead to the identification of novel therapeutic targets with high translational potential.

Protein phosphatases, including PP1, PP2A, and the dual-specificity phosphatase (DUSP) family, regulate MAPK signaling by dephosphorylating serine/threonine residues [19, 20]. Loss of phosphatase activity is a key non-canonical mechanism driving sustained MAPK activation and tumor progression [19, 20]. While PP1/PP2A and DUSPs both dephosphorylate ERK1/2, they have distinct functions: PP2A targets both ERK1 and ERK2 [21], whereas DUSPs primarily act on ERK2 [22, 23]. However, the molecular factors regulating phosphatase activity and their role in MAPK signaling remain largely unclear. Notably, the question of whether lncRNA-derived microproteins modulate phosphatase activity to impact MAPK signaling remains entirely unexplored. Investigating this potential non-canonical pathway is crucial, as it could uncover novel regulatory mechanisms in MAPK activation and provide insights into new therapeutic strategies for tumor progression.

In this study, we identify a novel group of lncRNAs in HCC with translational potential and demonstrate that *ASH1L-AS1* encodes the microprotein APPLE, which contributes to HCC progression by sustaining MAPK pathway activation. Mechanistically, we show that *ASH1L-AS1* - encoded APPLE directly interacts with p-ERK1/2 and the phosphatases PP1/PP2A to impacting ERK1/2 dephosphorylation. Furthermore, we reveal that the transcription factor E2F1 binds to the *ASH1L-AS1* promoter, upregulating its transcription and increasing APPLE expression. This in turn enhances ERK1/2 phosphorylation and accelerates HCC progression. Drug sensitivity analysis highlights the therapeutic potential of targeting the E2F1–ASH1L-AS1/APPLE–ERK1/2 axis. This study is the first to link E2F1, *ASH1L-AS1*, APPLE, PP1/PP2A, and MAPK signaling, challenging the traditional Ras-Raf-MEK model of MAPK regulation. Our findings uncover a non-canonical regulatory mechanism, offering new insights into MAPK hyperactivation in HCC and paving the way for innovative therapeutic strategies.

## Methods

### Discovery of translatable lncRNA associated with HCC

The TCGA-LIHC dataset was downloaded from the TCGA GDC data portal (https://portal.gdc.cancer.gov/), and the samples were divided into tumor and normal adjacent groups. Differential expression analysis was performed using the limma package, selecting genes with a fold change (FC) > 1.5 and *p* < 0.01 as significantly different. Translatable lncRNA annotations were annotated by the TransLnc database, and an intersection analysis was conducted with the DEGs to identify translatable lncRNAs related to HCC. The subcellular localization of proteins encoded by these translatable lncRNAs was analyzed using DeepLoc 2.0 [24]. Finally, the relationship between these HCC-related translatable lncRNAs and clinical variables were systematically analyzed, including tumor stage, prognosis, age, gender, estrogen signaling, lymph node involvement, metastasis, and race.

### Immune cell infiltration estimation and tumor immune phenotype classification

To investigate the relationship between *ASH1L-AS1* expression and the immune microenvironment in HCC, we utilized the xCell algorithm [25] to estimate the relative enrichment scores of various stromal and immune cell types based on bulk RNA-seq data from the TCGA-LIHC dataset. The predicted cell infiltration levels were then correlated with *ASH1L-AS1* expression using Pearson correlation analysis, and visualized using the corrplot R package. To further classify tumors into immune “hot” or “cold” phenotypes, we calculated immune activity scores using the GSVA (Gene Set Variation Analysis) R package [26]. Specifically, a gene set comprising *CD8A*, *CD8B*, *IFNG*, *CXCL9*, *CXCL10*, *GZMA*, *IDO1*, *LAG3*, *PDCD1*, and *CD274* was used to evaluate immune activation. Based on the median GSVA score, patients were dichotomized into immune hot tumors (high immune score) and cold tumors (low immune score). The expression levels of *ASH1L-AS1* were then compared between these two groups to assess its association with tumor immune phenotype.

### Somatic mutation analysis

Somatic single nucleotide variant (SNV) data for HCC patients were retrieved from the TCGA-LIHC cohort. Following quality control and preprocessing, the mutational profiles were analyzed to identify frequently mutated genes. A mutation landscape was subsequently visualized using the ComplexHeatmap R package (version 2.16.0), generating a waterfall plot to highlight the distribution and frequency of somatic alterations across individual tumor samples.

### Cell culture, tissue collection, and SCH772984 treatment

HepG2 and Huh7 HCC cell lines were purchased from ATCC and cultured in DMEM (Gibco, Cat. No.: C11995500BT, Grand Island, NY, USA) supplemented with 10% FBS (Gibco, Cat. No.: 10099–141 C, Grand Island, NY, USA) and 1% penicillin-streptomycin (Gibco, Cat. No.: 15140-122, Grand Island, NY, USA). Tumor tissues and matched adjacent normal tissues were collected from five hepatocellular carcinoma patients at the First Affiliated Hospital of Guangzhou Medical University. This study was approved by the Ethics Committee of Guangzhou Medical University (No.: 202412026), and all participants provided written informed consent. For SCH772984 treatment, a 1 M stock solution was prepared in dimethyl sulfoxide (DMSO) (Solarbio, Cat. No.: D8371, Beijing, China), and cells were treated with 20 µM SCH772984 (MCE, New Jersey, USA) for the specified durations.

### Stable cell line construction

Lentiviral vectors carrying *ASH1L-AS1* 5’-UTR-ORF-Flag, its translation initiation codon mutant (5’-UTR-ORFmut-Flag), and two specific shRNAs targeting *ASH1L-AS1* (shASH1L-AS1-#1, shASH1L-AS1-#2) were synthesized by Nanjing Zebrafish Biotech Co.,Ltd. HepG2 and Huh7 cells were infected with the lentiviral vectors to establish stable cell lines, including overexpression constructs (Normal control (NC), 5’-UTR-ORF-Flag, 5’-UTR-ORFmut-Flag) and shRNA constructs (shASH1L-AS1-#1, shASH1L-AS1-#2, shRNA control (shNC)). Stable cells were selected with puromycin for 2–3 weeks. Western blot (WB) and quantitative PCR (qPCR) were used to confirm overexpression and knockdown efficiency.

### CCK-8 and EdU assays

Cells were seeded in 96-well plates, and 10 µL of CCK-8 reagent was added at 1 day, 2 days, 3 days, and 4 days post-seeding. Absorbance at 450 nm was measured using a SpectraMax i3x microplate reader (Molecular Devices, San Jose, CA, USA). Each experimental group consisted of five biological replicates. For EdU assay, EdU incorporation was detected using the Cell-Light™ EdU Apollo In Vitro Kit (Ribobio, Guangzhou, China) according to the manufacturer’s protocol, and the percentage of EdU-positive cells was calculated. Five random fields from each well were selected and analyzed under a fluorescent microscope (Olympus, Tokyo, Japan).

### Wound healing assay

Cells were seeded in 6-well plates and cultured to confluence. A uniform wound was created by a culture-insert (Ibidi, Martinsried, Germany). After 1 × PBS washing, cells were cultured in complete medium, and images were captured at 0 h and 24 h post-scratching using a phase-contrast microscope (Olympus, Tokyo, Japan). The wound closure was quantified by measuring the remaining gap using ImageJ software. For each condition, five random fields were selected for statistics analysis.

### Transwell invasion assay

Invasion was assessed using Transwell chambers (Corning, NY, USA) with 8 μm pore size. The stable cells were seeded in the upper chamber with serum-free medium, and the lower chamber contained medium with 10% FBS as a chemoattractant. After 24 h, invading cells were fixed with 4% paraformaldehyde, stained with DAPI (Beyotime, Shanghai, China). The number of invaded cells was counted in five randomly selected fields under a fluorescent microscope (Olympus, Tokyo, Japan). Images were captured, and the invasive cells were quantified.

### Generation of APPLE-specific polyclonal antibody

To facilitate the detection of the endogenous APPLE microprotein, a custom APPLE-specific polyclonal antibody was designed and generated by GenScript (China). The antigenic peptide was selected based on the unique amino acid sequence of APPLE to ensure specificity, and the antibody was raised in rabbits following standard immunization protocols. The resulting antiserum was affinity-purified and validated for specificity through WB and immunofluorescence (IF) assays.

### Immunofluorescence staining

Cells were incubated overnight at 4 °C with primary antibodies: anti-Flag (Sigma Aldrich, Cat. No.: F1804, 1:500 dilution) and anti-p-ERK1/2 (Proteintech, Cat. No.: 28733-1-AP, 1:500 dilution), anti- PPP2R2A (Proteintech, Cat. No.: 16569-1-AP, 1:100 dilution), anti- MYPT1 (Proteintech, Cat. No.: 22117-1-AP, 1:500 dilution), and anti- PPP1CB (Proteintech, Cat. No.: 10140-2-AP, 1:500 dilution). After washing, cells were incubated with Alexa Fluor 594-conjugated secondary antibodies (CST, Cat. No.: 8890, 1:2000 dilution) for 1 h at room temperature. GFP fusion proteins were observed directly under a fluorescent microscope. Nuclei were counterstained with DAPI, and images were captured using a fluorescent microscope (Olympus, Tokyo, Japan).

### Western blot

Total protein was extracted from cells and tissues using the RIPA Lysis Buffer (Beyotime, Shanghai, China) according to the manufacturer’s protocol. For Western blot (WB), protein samples were separated by SDS-PAGE and transferred to PVDF membranes. The membranes were incubated overnight at 4 °C with primary antibodies for 1 h at room temperature. Protein bands were visualized and quantified with the Odyssey system. The details of the primary antibodies were listed in Table S1.

### Bulk RNA-Seq analysis

Cell pellets were collected and lysed with TRIzol reagent (Invitrogen, California, USA), then sent to Igenebook Biotechnology (Wuhan, China) for routine bulk RNA sequencing. The resulting gene expression matrix was analyzed using R software (R software, version 4.3.3). Principal component analysis (PCA), clustering analysis, and differential gene expression analysis were performed using the FactoMineR, limma, and pheatmap R packages. Volcano plots were generated to visualize differentially expressed genes (DEGs). Gene Ontology (GO) and Kyoto Encyclopedia of Genes and Genomes (KEGG) functional enrichment analyses were carried out using the ClusterProfile R package to identify enriched biological functions and pathways.

### Xenograft tumor assay

Xenograft tumor implantation was performed in 5-week-old Balb/c nude mice to evaluate the effect of *ASH1L-AS1* encoding the microprotein APPLE on tumorigenesis. The nude mice (5 per group) were divided into three groups: NC, 5’-UTR-ORF-Flag, and 5’-UTR-ORFmut-Flag. Tumor cells (5 × 10⁶ cells) were subcutaneously injected. Tumor volume was measured weekly using calipers, with volume calculated as Volume = (length × width²) / 2. After 4 weeks, tumors were excised, weighed, and compared between groups. All procedures were approved by the Institutional Animal Care and Use Committee of Guangzhou Medical University (No: GB2024-003).

### qRT-PCR assay

Total RNA was extracted from cells using Foregene RNA isolation kit (Foregene, Chengdu, China) following the manufacturer’s protocol. cDNA was synthesized from the RNA using TOYOBO ReverTra Ace^®^ qPCR RT Master Mix (Osaka, Japan), according to the provided instructions. Then qPCR was performed using the PowerUP™ SYBR™ Green Master Mix (Thermo Fisher Scientific, Waltham, MA, USA). The primers for the target genes were detailed in Table S2.

### Co-immunoprecipitation and mass apectrometry analysis

Stable HepG2 cells overexpressing APPLE-Flag and control cells transfected with empty vector were established. Co-immunoprecipitation (Co-IP) was performed using anti-Flag antibody (Sigma Aldrich, Cat. No.: F1804, 1:500 dilution) to isolate interacting proteins from both cell lines. The immunoprecipitated products were separated by SDS-PAGE and visualized by silver staining to assess Co-IP efficiency and identify differential protein bands between APPLE-Flag and control groups. Co-IP samples were subsequently sent to Beijing Novogene Co., Ltd. for 3D label-free proteomic analysis to identify APPLE-specific interacting proteins.

To validate the interaction between APPLE and ERK1/2, WB was performed using ERK1/2-specific antibodies (Proteintech, Cat. No. 11257-1-AP, 1:2000 dilution) on Co-IP products derived from APPLE-Flag-expressing cells. Additionally, a series of APPLE truncation mutants (Flag-tagged) were constructed and overexpressed in HepG2 cells, including MUT1 (deletion of amino acids 21–40), MUT2 (deletion of 41–60), and MUT3 (deletion of 61–80). Co-IP was conducted using anti-Flag antibody, and the binding of each mutant to ERK1/2 was analyzed to map the functional interaction domain.

### Chromatin immunoprecipitation followed by quantitative PCR

To examine transcript factor E2F1 binding to the *ASH1L-AS1* promoter, Chromatin Immunoprecipitation followed by quantitative PCR (ChIP-qPCR) was performed. Briefly, cells were crosslinked with 1% formaldehyde for 10 min, quenched with glycine, and then lysed to isolate chromatin. Chromatin was fragmented to an average size of 200 bp to 1000 bp by sonication. Immunoprecipitation was performed using an anti-E2F1 antibody or an isotype control antibody (IgG), followed by incubation with protein A/G beads. After washing and elution, crosslinking was reversed at 62 °C for 2 h, and the DNA was purified. Moreover, qPCR was performed on the precipitated DNA using primers targeting the *ASH1L-AS1* promoter region. Relative enrichment of the *ASH1L-AS1* promoter in E2F1-immunoprecipitated samples was calculated using the 2^−ΔΔCt^ method, with input DNA as a control. For comparison, IgG-immunoprecipitated regions were used as a negative control to assess non-specific binding.

### Luciferase reporter assay

The *ASH1L-AS1* promoter region, identified in the ChIP-qPCR as a potential E2F1 binding site, was cloned into the pGL3-basic luciferase vector. A mutant promoter construct, with the key E2F1 binding sites disrupted, was also created as a negative control. HEK 293T cells were transfected with either the wild-type or mutant promoter-luciferase reporter constructs, along with a Renilla luciferase plasmid for normalization. E2F1 overexpression was performed using the corresponding plasmids. After 24–48 h, luciferase activity was measured using the Dual-Luciferase Reporter Assay System, and the firefly luciferase activity was normalized to renilla luciferase activity. The relative luciferase activity of the wild-type reporter was compared with the mutant construct to confirm the role of E2F1 binding in regulating *ASH1L-AS1* promoter activity.

### Drug sensitivity analysis

OncoPredict [27] was used to assess the drug sensitivity of TCGA-LIHC patient samples based on their gene expression profiles. This analysis provides predictions of therapeutic efficacy for various drugs based on individual genetic signatures. Additionally, drug sensitivity analysis was performed using the Cancer Therapeutics Response Portal 2 (CTRP2) [28] (https://portals.broadinstitute.org/ctrp.v2.1/), which offers a platform for evaluating the response of cancer cell lines to a range of targeted and chemotherapeutic agents. Data from the CTRP2 database were utilized to further analyze the correlation between gene expression profiles and drug sensitivity in HCC.

### Statistical analysis

Statistical analyses were conducted using R software (R software, version 4.3.3) and GraphPad Prism 9 (GraphPad Software, Inc., La Jolla, CA). The following statistical tests were applied: paired Student’s t-test, multiple t-test, one-way and two-way ANOVA, Wilcoxon test, and log-rank test, as appropriate. Sample sizes (n) are provided in the figure legends. Statistical significance was defined as *P < 0.05, **P < 0.01, and ***P < 0.001 for all analyses.

## Results

### Comprehensive transcriptome-wide discovery of the translatable LncRNAs associated with HCC

**Fig. 1:**
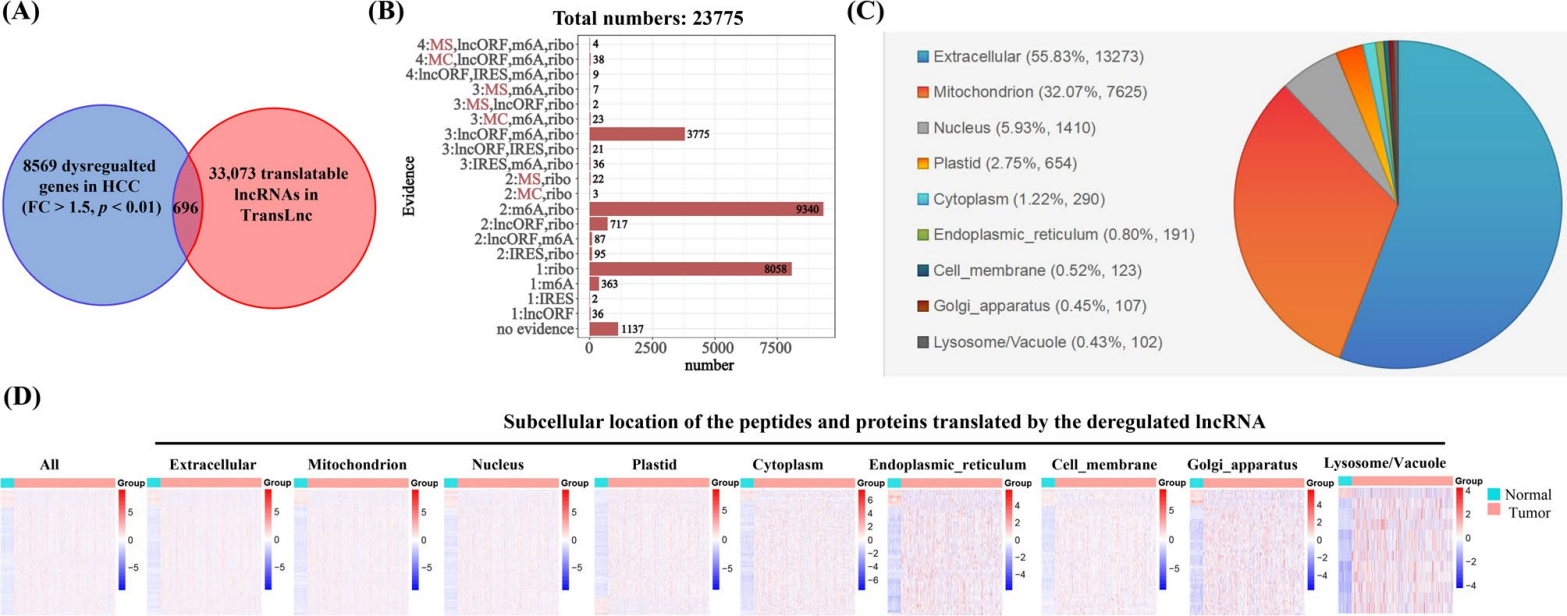
Identification of potential translatable lncRNAs associated with HCC. **(A)** Identification of potential translatable lncRNAs by intersecting differentially expressed genes (|Fold Change (FC)| ≥ 1.5, p-value ≤ 0.01) with the TranLnc database. **(B)** Distribution of these translatable lncRNAs based on different types of translational evidence. **(C)** Subcellular localization of proteins encoded by translatable lncRNAs. **(D)** Heatmap depicting the expression patterns of translatable lncRNAs, categorized by the subcellular localization of their encoded products

To discover the translatable lncRNAs associated with HCC, we firstly analyzed the TCGA-LIHC dataset to identify DEGs in HCC tissues compared with adjacent normal tissues (|Fold Change (FC)| ≥ 1.5 and p-value ≤ 0.01) (Fig. 1A). By intersecting these DEGs with 33,073 translational lncRNAs from the TransLnc database [29], we identified 696 differentially expressed translatable lncRNAs (Fig. 1A). These lncRNAs produced 2,777 transcripts, potentially encoding 23,775 ncORFs, with experimental evidences of mass spectrometry (MS), manual curation (MC), ribo-seq, m6A, and others (Fig. 1B). Among these, 95.06% (22,600/23,775) of the ncORFs showed ribo-seq or m6A-related evidence, and 99 ncORFs have high-confidence evidences of MS or MC (Fig. 1B).

Given that protein subcellular localization is closely linked to its function, we utilized the DeepLoc tool to predict subcellular localization of the microproteins encoded by these lncRNA-derived ncORFs. In line with previous studies [30], these microproteins predominantly localize to extracellular (55.86%), mitochondria (32.07%), nucleus (5.96%), plastid (2.75%), and cytoplasm (1.22%) (Fig. 1C). Moreover, heatmap analysis further revealed significant differences in the expression of translatable lncRNAs between HCC tissues and adjacent normal tissues, irrespective of their subcellular localization (Fig. 1D). This implies that the microproteins encoded by lncRNAs may have functional specificity and could be involved in the regulation of cellular functions. Additionally, 24 translatable lncRNAs associated with HCC, supported by high-confidence translational evidence, were identified (Table 1). Overall, these results underscore the widespread association of translatable lncRNAs with HCC, highlighting their potential functional roles and their value as biomarkers for the malignancy.

**Table 1:** Twenty-four translatable LncRNAs associated with HCC supported by high-confidence evidence

Translatable lncRNAs	Normal vs. Tumor	Survival	Clinical stage	T stage	*N* stage	M stage	Peptide length	Translatable evidence
CTBP1-DT	**** (↑)	–	–	–	–	–	93aa	MS, lncORF, m6A, ribo
ASH1L-AS1	**** (↑)	*	**	**	–	–	90aa	MC, lncORF, m6A, ribo
FGF14-AS2	**** (↓)	–	–	–	–	–	74aa	MC, lncORF, m6A, ribo
NEAT1	**** (↑)	–	–	–	–	*	71aa	MC, lncORF, m6A, ribo
AC009237.14	**** (↑)	–	–	–	–	–	67aa	MC, m6A, ribo
LINC01702	**** (↓)	–	*	*	*	–	67aa	MS, ribo
FOXD2-AS1	**** (↑)	***	–	–	–	–	64aa	MS, m6A, ribo
MIR34AHG	**** (↑)	–	–	–	–	*	64aa	MS, lncORF, m6A, ribo
PSMB8-AS1	**** (↑)	–	–	–	–	–	63aa	MC, lncORF, m6A, ribo
SCAMP1-AS1	**** (↑)	*	–	–	–	–	61aa	MC, lncORF, m6A, ribo
LINC01018	**** (↓)	–	**	**	–	–	57aa	MS, ribo
DLG5-AS1	**** (↑)	–	–	–	–	–	54aa	MS, ribo
GPRC5D-AS1	**** (↑)	–	–	–	–	–	49aa	MS, lncORF, ribo
LINC01278	**** (↑)	–	–	*	–	–	49aa	MS, ribo
TSPEAR-AS2	**** (↑)	–	–	–	–	–	48aa	MS, lncORF, m6A, ribo
OTUD6B-AS1	**** (↑)	–	–	*	–	–	44aa	MS, ribo
CRNDE	**** (↑)	–	–	*	–	–	43aa	MC, lncORF, m6A, ribo
LINC01488	**** (↓)	–	***	****	–	–	39aa	MS, ribo
SNHG8	**** (↑)	–	–	–	–	–	36aa	MC, lncORF, m6A, ribo
RAB30-DT	**** (↑)	**	*	**	–	–	32aa	MC, lncORF, m6A, ribo
ZFAS1	**** (↑)	**	*	*	–	–	25aa	MC, lncORF, m6A, ribo
GIHCG	**** (↑)	***	**	**	–	–	24aa	MC, lncORF, m6A, ribo
SNHG1	**** (↑)	–	*	*	–	–	21aa	MS, lncORF, ribo
LINC00294	**** (↑)	–	–	–	–	*	18aa	MS, m6A, ribo

*: *p* ≤ 0.05; **: *p* ≤ 0.01; ***: *p* ≤ 0.001; ****: *p* ≤ 0.0001. MS: Mass spectrometry evidence; MC: Manually curated; lncORF: Contains a predicted open reading frame (ORF) within the lncRNA; m6A: Exhibits RNA N6-methyladenosine (m6A) modification; Ribo: Supported by ribosome profiling evidence

### *ASH1L-AS1* associates with HCC progression and poor prognosis

**Fig. 2:**
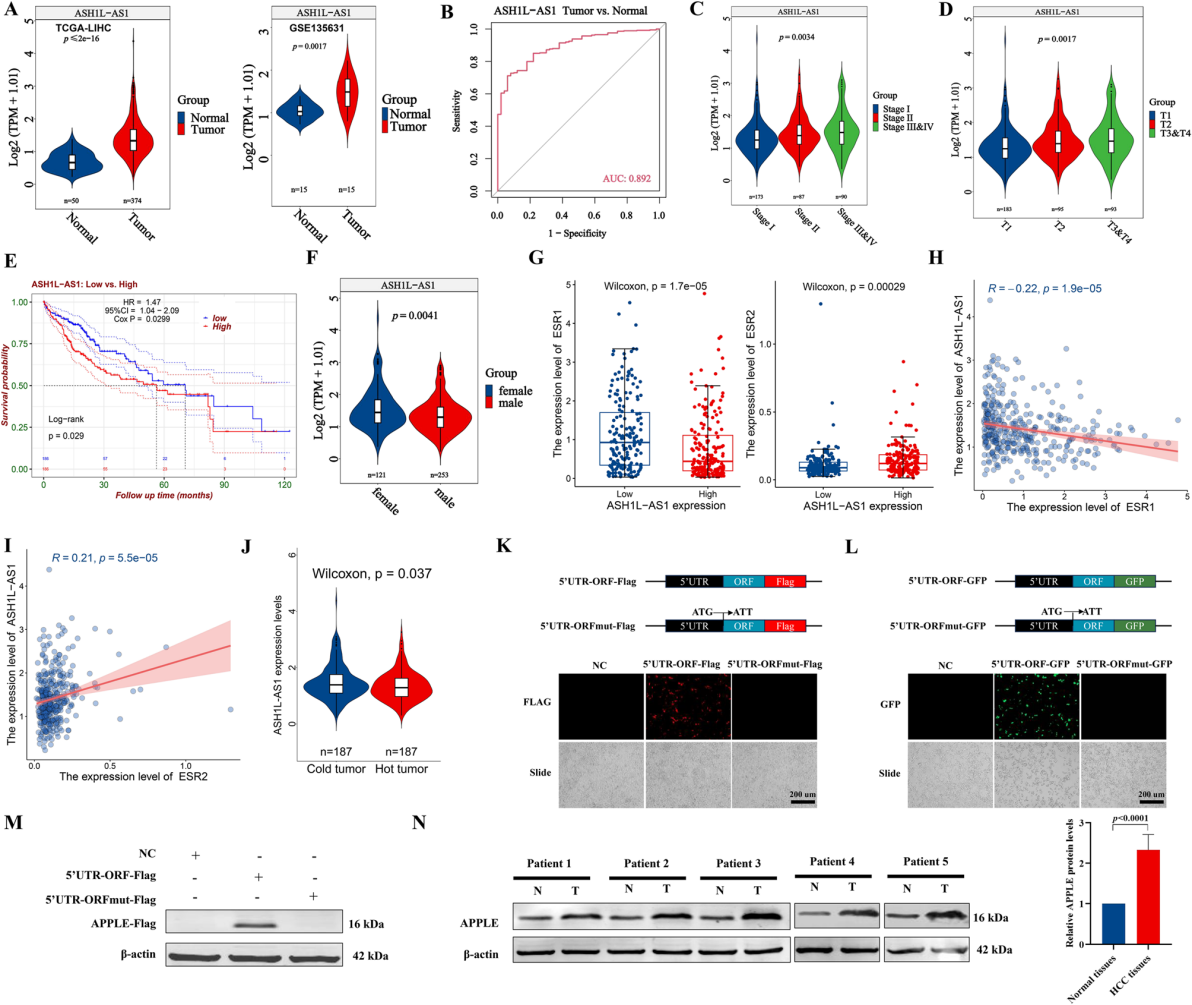
*ASH1L-AS1* encodes the microprotein APPLE and is closely associated with HCC progression. **(A)** *ASH1L-AS1* is significantly upregulated in HCC tissues. **(B)** ROC analysis indicating that *ASH1L-AS1* serves as an independent diagnostic factor for HCC. **(C-D)** Gene expression levels of *ASH1L-AS1* progressively increase from early to late stages of HCC. **(E)** Kaplan-Meier survival analysis showing that high *ASH1L-AS1* expression is significantly associated with poor prognosis in HCC patients. **(F)** Violin plots illustrating the correlation between *ASH1L-AS1* expression and sex. **(G)** Boxplot showing the expression levels of *ESR1* and *ESR2* in patients with high and low *ASH1L-AS1* expression. **(H-I)** Correlation analysis between *ASH1L-AS1* expression and *ESR1*/*ESR2* gene expression levels. **(J)** Differential expression of *ASH1L-AS1* between immune cold and hot tumors. **(K-L)** Immunofluorescence analysis demonstrating that APPLE can be overexpressed in HepG2 cells through Flag- and GFP-tagged fusion constructs. **(M)** Western blot analysis confirming APPLE expression in HepG2 cells using Flag fusion tags. **(N)** Western blot analysis using an APPLE-specific antibody revealing a significant upregulation of endogenous APPLE protein in HCC tissues

In the 24 translatable lncRNAs, *ASH1L-AS1* expression was significantly higher in HCC tumor tissues compared with adjacent normal tissues (Fig. 2A) and effectively distinguished tumor from normal samples (Fig. 2B). Expression of *ASH1L-AS1* increased progressively from early to late stages of HCC (Fig. 2C&D and Fig. S1A&B), and was significantly associated with poor survival prognosis (Fig. 2E). *ASH1L-AS1* expression was higher in female patients than in male patients (Fig. 2F), but no significant associations were found with age or ethnicity (Fig. S1C&D). Considering the observed sex-based differences in *ASH1L-AS1* expression, further analysis revealed that *ESR1* expression was significantly downregulated in patients with high *ASH1L-AS1* expression, showing a strong negative correlation between the two (Fig. 2G&H). In contrast, *ESR2* expression was significantly upregulated and exhibited a positive correlation with *ASH1L-AS1* expression (Fig. 2G&I). These findings are consistent with recent studies, where *ESR1* was reported to suppress liver carcinogenesis [31], while *ESR2* gene polymorphisms (e.g., rs2978381) were shown to significantly increase the risk of HBV-related liver cirrhosis and HCC [32].

Moreover, we further investigated the association between aberrant *ASH1L-AS1* expression and immune cell infiltration in HCC tumors. The analysis revealed that *ASH1L-AS1* expression was significantly negatively correlated with the infiltration of CD8⁺cytotoxic T cells and macrophages, while showing positive correlations with CD4⁺T memory cells, Th1/Th2 cells, and gamma delta T cells—representing helper or non-cytotoxic immune subsets (Fig. S1E). These suggest that high *ASH1L-AS1* expression is associated with a “cold tumor” immune phenotype. Despite some activity from Th1/Th2 and gamma delta T cells, the lack of CD8⁺T cell infiltration may lead to impaired antitumor immune responses. To further confirm this, we performed immune hot/cold tumor classification based on GSVA-derived immune scores from key immune activation genes. This analysis demonstrated that *ASH1L-AS1* expression was significantly upregulated in immune cold tumors (Fig. 2J), corroborating the above observations.

Although higher *ASH1L-AS1* expression was observed in patients with tumor metastasis and lymph node involvement, these differences did not reach statistical significance due to the small sample size (Fig. S1F&G). Further analysis of data from 1380 HCC patients across 5 studies in cBioPortal [33] revealed that 6% of patients exhibited *ASH1L-AS1* amplification and structural variations (Fig. S2A), suggesting that its upregulation may involve other unrevealing mechanisms. Additionally, upregulated *ASH1L-AS1* was found to be associated with higher mutation frequencies in hotspot genes in HCC (Fig. S2B). However, no significant difference was observed in the mutation frequency of the oncogenic *RAS* genes between the high and low *ASH1L-AS1* expression groups, which is consistent with its relatively low mutation rate in HCC (approximately 3%) (Fig. S2C). *ASH1L-AS1* expression did not significantly differ between RAS-mutant and wild-type HCC patients (Fig. S2D). These results suggest that *ASH1L-AS1* could serve as a diagnostic and prognostic marker for HCC.

### *ASH1L-AS1* encodes the microprotein APPLE to promote tumorigenesis in HCC

To verify whether *ASH1L-AS1* encodes the microprotein APPLE, we conducted in vitro overexpression experiments, and the results showed that *ASH1L-AS1* stably expresses APPLE microprotein in HCC cells (Fig. 2K-M). Additionally, a polyclonal antibody specifically targeting APPLE was generated to confirm the expression of endogenous microprotein APPLE. Using this antibody, WB analysis of five pairs of HCC tumor and adjacent normal tissues detected the expected ~ 15 kDa APPLE microprotein, which was significantly upregulated in HCC tumors compared with normal tissues (Fig. 2N). These findings suggest that *ASH1L-AS1* may regulate HCC progression by encoding the microprotein APPLE.

**Fig. 3:**
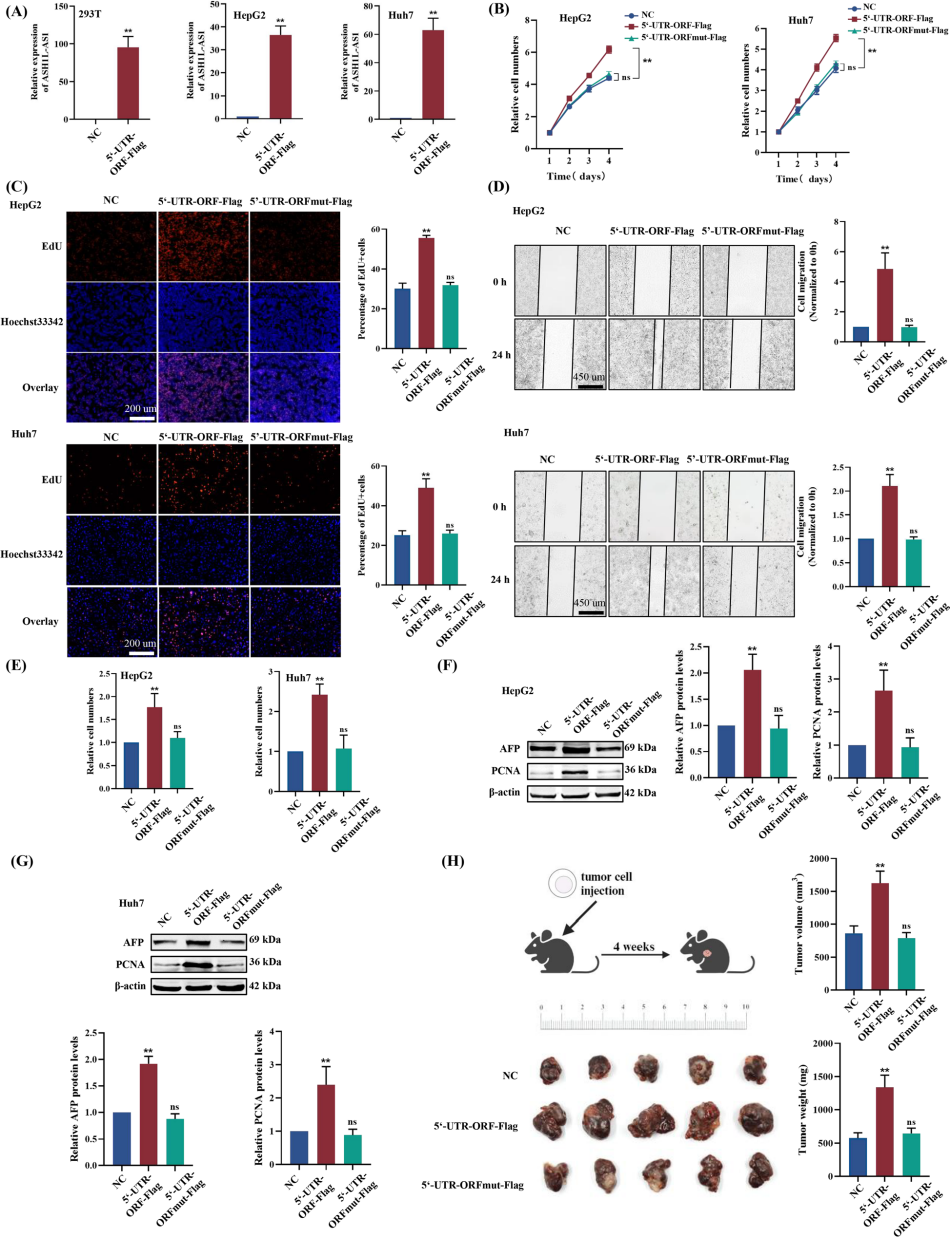
Overexpression of the microprotein APPLE encoded by *ASH1L-AS1* promotes HCC progression. **(A)** qPCR validation of stable APPLE overexpression. **(B-E)** CCK-8 assay **(B)**, EdU staining assay **(C)**, wound healing assay **(D)**, and Transwell assay **(E)** demonstrating that APPLE overexpression enhances the proliferation, migration, and invasion of HepG2 and Huh7 cells. **(F-G)** Western blot analysis showing that APPLE overexpression upregulates AFP and PCNA protein expression. **(H)** Xenograft tumor model in nude mice indicating that APPLE overexpression enhances the tumorigenic capacity of HepG2 cells in vivo

**Fig. 4:**
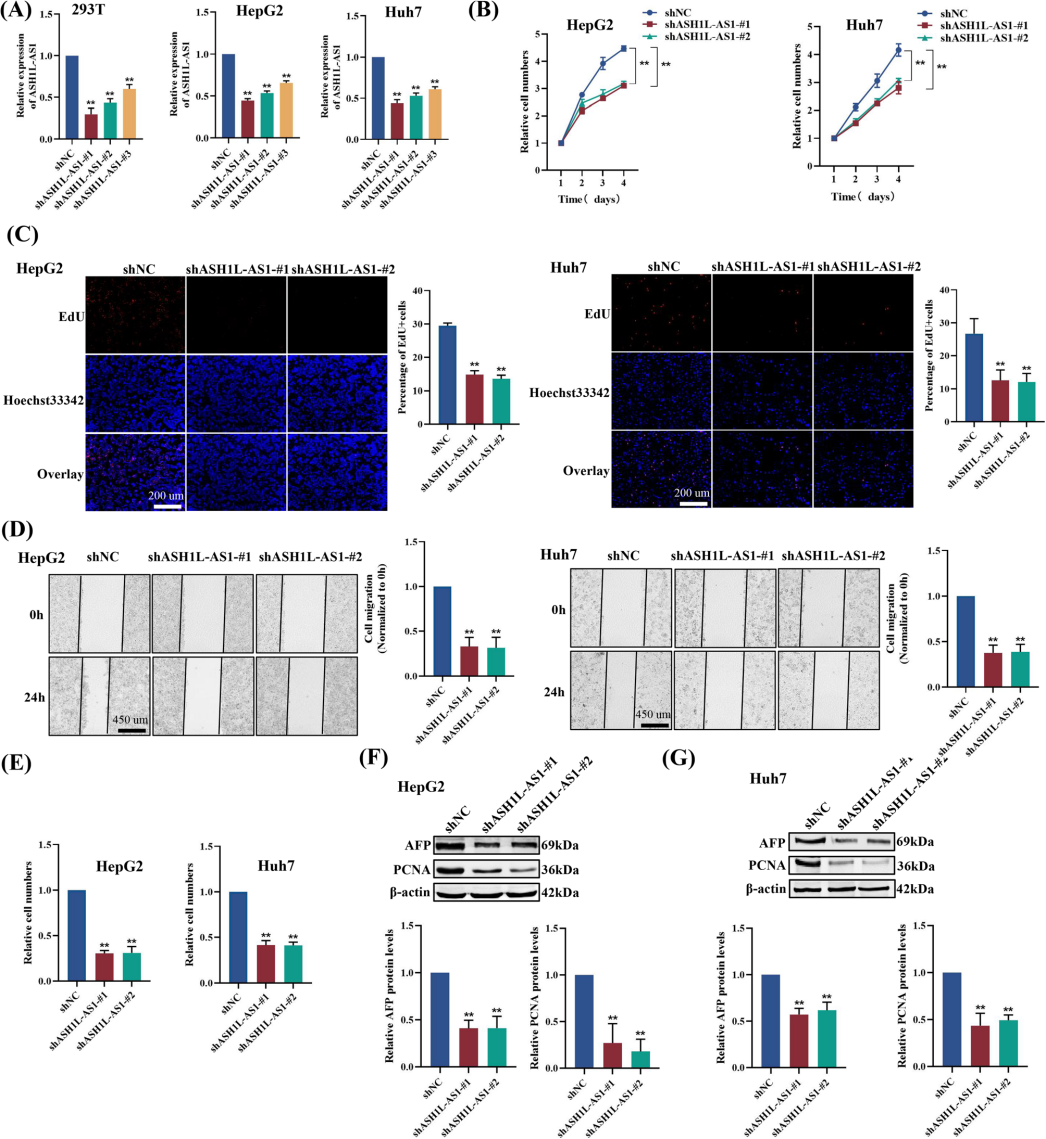
Knockdown of *ASH1L-AS1* suppresses HCC progression. **(A)** qPCR validation of stable *ASH1L-AS1* knockdown. **(B-E)** CCK-8 assay **(B)**, EdU staining assay **(C)**, wound healing assay **(D)**, and Transwell assay (**E**) showing that *ASH1L-AS1* knockdown inhibits the proliferation, migration, and invasion of HepG2 and Huh7 cells. **(F-G)** Western blot analysis demonstrating that *ASH1L-AS1* knockdown reduces AFP and PCNA protein expression

In order to investigate the role of APPLE in HCC, we established stable HepG2 and Huh7 cell lines overexpressing APPLE (5‘-UTR-ORF-Flag) or a start codon-mutated version (5’-UTR-ORFmut-Flag), as well as *ASH1L-AS1* knockdown cell lines. Overexpression of APPLE in HepG2 and Huh7 cells significantly enhanced cell proliferation (Fig. 3A-C), migration (Fig. 3D), invasion (Fig. 3E), tumor biomarker levels (AFP and PCNA, Fig. 3F&G), and tumorigenic ability in vitro and in vivo (Fig. 3H) compared to control cells. In contrast, cells overexpressing the start codon-mutated version did not exhibit these significant changes (Fig. 3B-G). These results suggest that APPLE overexpression—rather than the RNA itself—promotes HCC progression by significantly increasing the expression levels of diagnostic biomarkers AFP and PCNA. Consistent with the overexpression results, APPLE knockdown led to decreased cell proliferation (Fig. 4A-C), migration (Fig. 4D), invasion (Fig. 4E), and reduced the AFP and PCNA levels (Fig. 4F&G). Collectively, these findings demonstrate that *ASH1L-AS1* promotes HCC progression by encoding the microprotein APPLE.

### The microprotein APPLE binds p-ERK1/2 and phosphatases PP1/PP2A to sustain MAPK activation for HCC progression

**Fig. 5:**
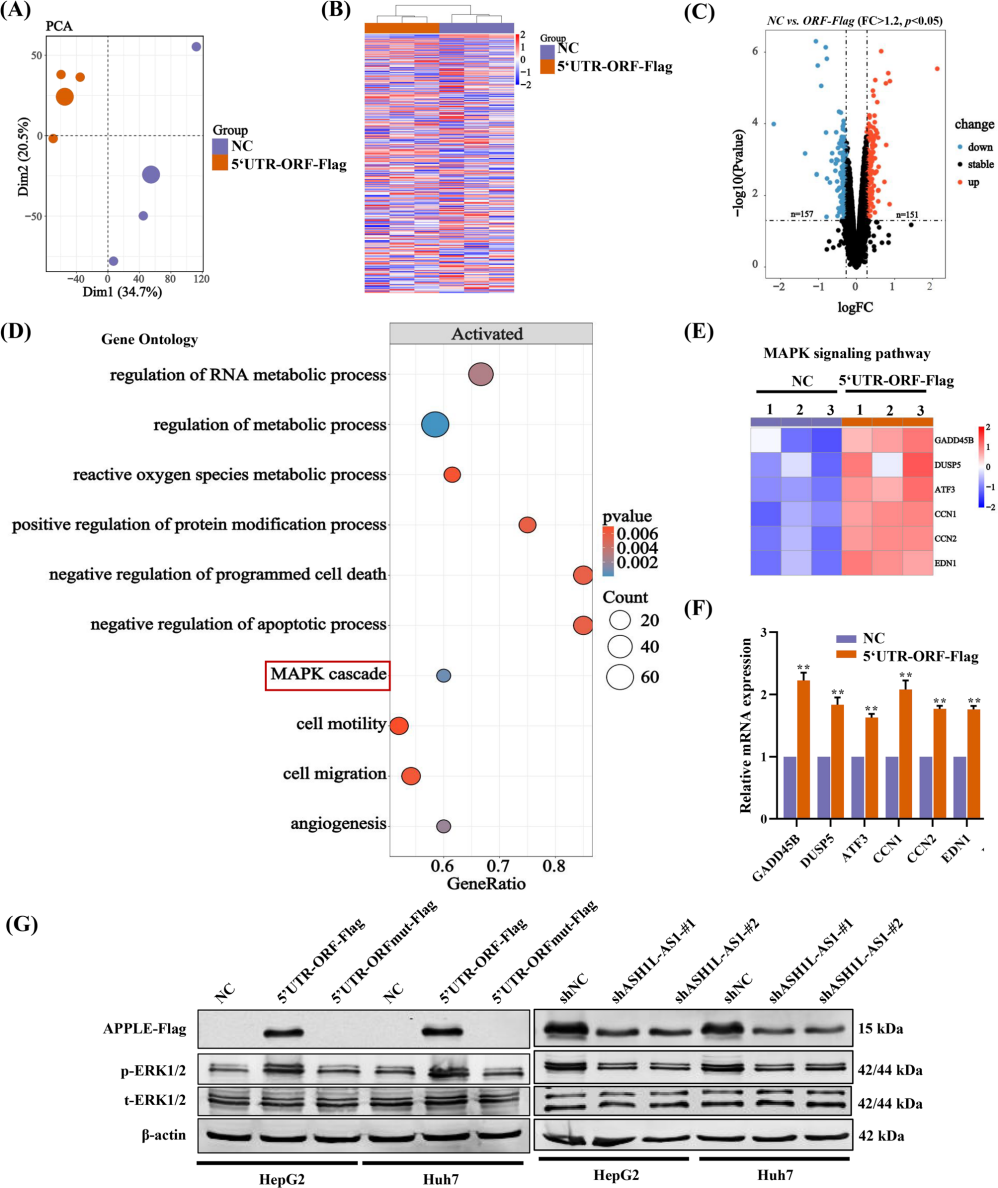
RNA-Seq analysis reveals that APPLE overexpression enhances the MAPK signaling in HCC cells. **(A-B)** PCA analysis **(A)** and heatmap **(B)** showing distinct gene expression profiles in HepG2 cells overexpressing APPLE (5’UTR-ORF-Flag) compared to normal controls (NC). **(C)** Volcano plot displaying the number of upregulated and downregulated genes in APPLE-overexpressing HepG2 cells. **(D)** Gene Ontology (GO) identifying functional pathways affected by APPLE overexpression. **(E)** Heatmap illustrating the differential expression patterns of genes enriched in the MAPK signaling pathway. **(F)** qPCR validation of differentially expressed genes involved in these pathways upon APPLE overexpression. **(G)** Western blot analysis demonstrating that APPLE overexpression significantly increases p-ERK1/2 levels, whereas its translation-incompetent codon mutant has no effect on p-ERK1/2 expression

To investigate the molecular mechanisms underlying APPLE’s regulation of HCC, RNA-Seq analysis was performed on HepG2 cells with or without APPLE overexpression (Fig. 5). This analysis revealed distinct gene expression profiles between the two cell lines (Fig. 5A&B) and identified 308 DEGs, with 151 upregulated and 157 downregulated (Fig. 5C). GO analysis showed that these DEGs were significantly involved in cell proliferation, migration, and invasion, with notable enrichment in MAPK signaling pathway (Fig. 5D). Subsequent heatmap and qPCR analyses confirmed the marked upregulation of genes enriched in this pathway, supporting that APPLE overexpression indeed activates MAPK signaling in HCC cells (Fig. 5E&F).

Similarly, HCC patients with high *ASH1L-AS1* expression displayed distinct gene expression patterns compared to those with low expression, with dysregulation of the similar key pathways, such as cell cycle and liver development (Fig. S3). WB analyses confirmed that overexpression of APPLE in HCC cells enhanced ERK1/2 phosphorylation and activation of associated pathways, whereas APPLE knockdown led to a reduction in ERK1/2 phosphorylation (Fig. 5G and Fig. S4). In contrast, cells overexpressing a start codon-mutated version of APPLE showed no significant change in ERK1/2 phosphorylation (Fig. 5G). These results strongly indicate that the *ASH1L-AS1*/APPLE axis drives HCC progression by activating the MAPK signaling pathway.

**Fig. 6:**
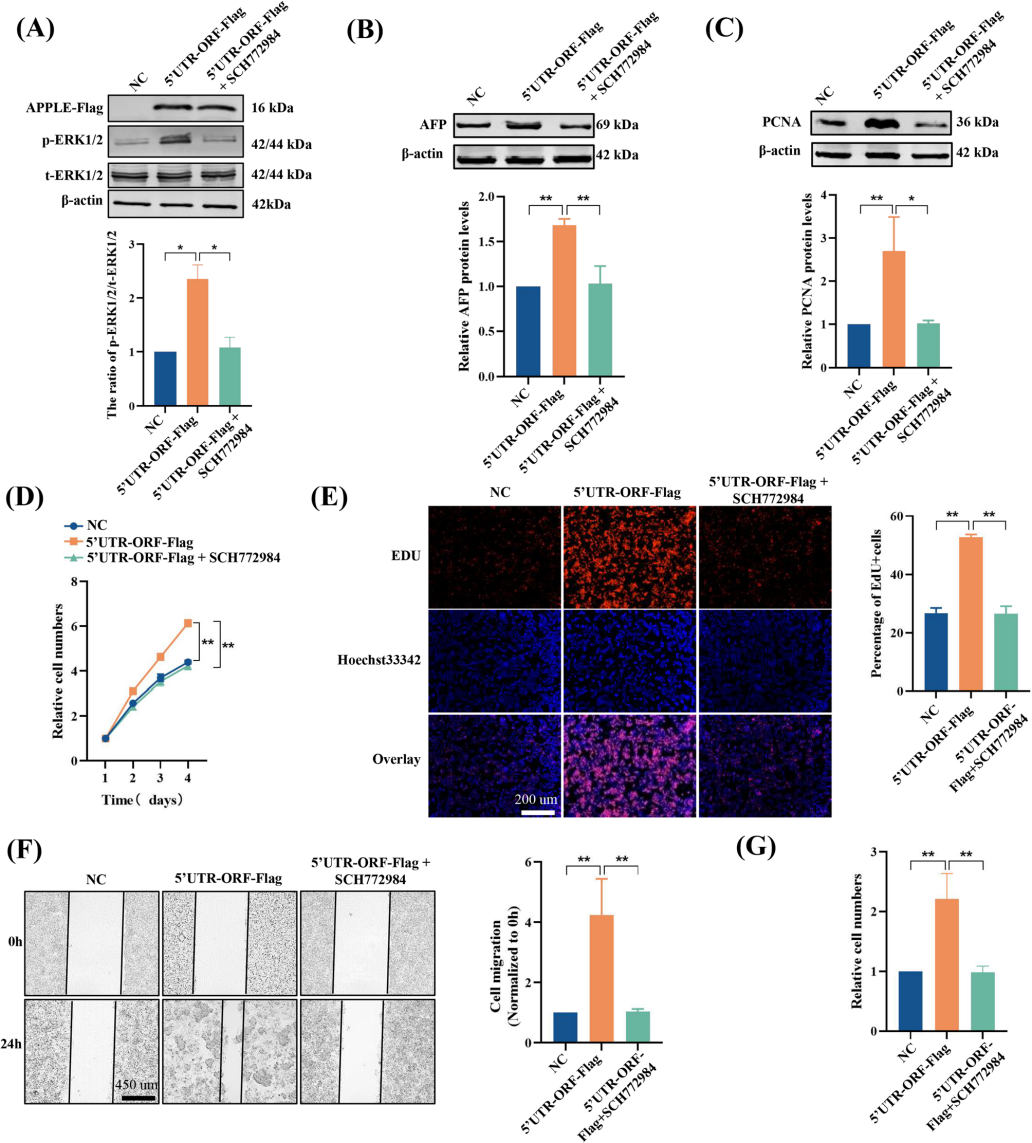
Inhibition of ERK1/2 reverses the oncogenic effects of APPLE overexpression in HCC cells. **(A-C)** Western blot analysis showing that SCH772984 treatment attenuates the APPLE overexpression-induced increase in p-ERK1/2 **(A)**, AFP **(B)**, and PCNA **(C)** protein levels in HepG2 cells. **(D-G)** CCK-8 **(D)**, EdU **(E)**, wound healing **(F)**, and Transwell **(G)** assays demonstrating that SCH772984 treatment reverses the APPLE overexpression-driven enhancement of HepG2 cell proliferation, migration, and invasion

To further validate this mechanism, we utilized the selective ERK1/2 inhibitor SCH772984 [34] in functional assays. Results showed that SCH772984 efficiently reversed APPLE-induced ERK1/2 phosphorylation (Fig. 6A), decreased the elevated expression levels of AFP (Fig. 6B) and PCNA (Fig. 6C), and inhibited APPLE-mediated proliferation, migration, and invasion of HCC cells (Fig. 6D-G). These results strongly confirm that the *ASH1L-AS1*/APPLE axis drives HCC progression via activation of the MAPK pathway.

**Fig. 7:**
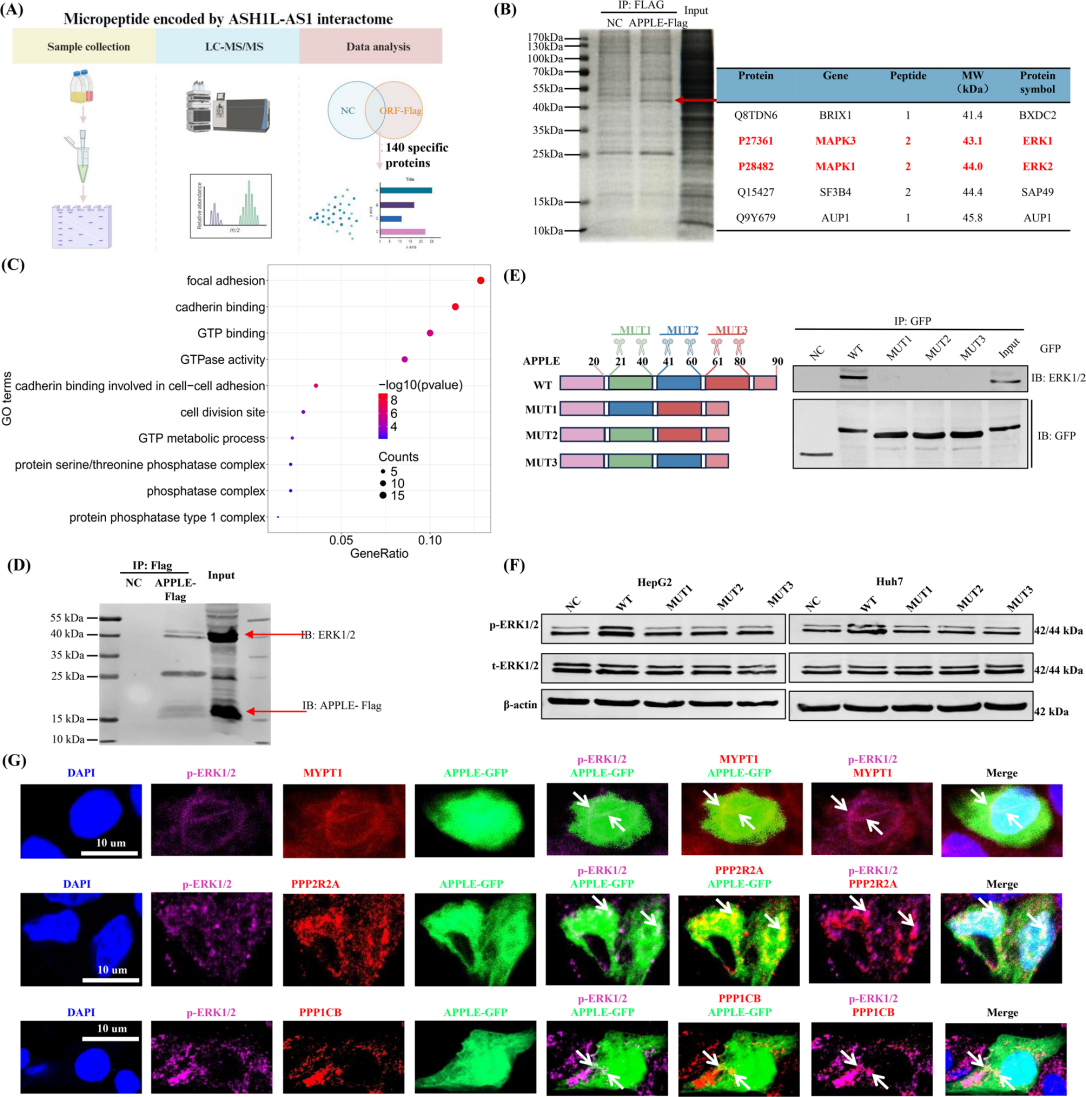
APPLE interacts with ERK1/2 and PP1/PP2A phosphatases to regulate ERK1/2 phosphorylation. **(A)** Schematic overview of Co-IP and mass spectrometry analysis used to identify APPLE-Flag interacting proteins. **(B)** Silver staining of Co-IP products using anti-Flag antibody in control and APPLE-Flag–overexpressing HepG2 cells. A specific ~ 43 kDa band was enriched in the APPLE-Flag group. **(C)** Gene Ontology (GO) enrichment analysis of APPLE-specific interacting proteins. **(D)** Western blot following Co-IP confirms the interaction between APPLE-Flag and ERK1/2. **(E)** Truncation mutant analysis demonstrates that full-length APPLE is required for binding ERK1/2. **(F)** Truncation mutants of APPLE fail to enhance ERK1/2 phosphorylation, indicating functional dependence on full-length APPLE. **(G)** IF analysis shows co-localization of APPLE-Flag with p-ERK1/2, as well as PP1 catalytic subunit PPP1CB, regulatory subunit PPP1R12A (MYPT1), and PP2A regulatory subunit PPP2R2A

To investigate the molecular mechanism by which APPLE increases ERK1/2 phosphorylation and activates the MAPK pathway, we performed Co-IP coupled with mass spectrometry in cells stably expressing Flag-tagged APPLE (Fig. 7A&B). A total of 140 APPLE-interacting proteins were identified (Table S3), with molecular weights predominantly ranging from 20 to 100 kDa (Fig. S5A). The genes encoding these proteins exhibited strong correlations in both expression and clinical relevance with *ASH1L-AS1* (Fig. S5B&C), further supporting the specificity of their interactions with APPLE-Flag.

Silver staining following Co-IP revealed a distinct ~ 43 kDa protein band enriched in the APPLE-Flag group compared to the negative control (NC) (Fig. 7B). Mass spectrometry analysis of this band identified five APPLE-Flag-specific interacting proteins, including ERK1 and ERK2 (Fig. 7B). GO analysis showed that these interacting proteins are significantly enriched in pathways related to the protein serine/threonine phosphatase complex and GTPase activity, suggesting that APPLE may modulate ERK1/2 phosphorylation to regulate MAPK signaling by interacting with and regulating phosphatase activity (Fig. 7C). The direct interaction between APPLE and ERK1/2 was further validated by Co-IP followed by WB (Fig. 7D). Notably, APPLE truncation mutants failed to bind ERK1/2 (Fig. 7E) and were unable to increase ERK1/2 phosphorylation (Fig. 7F), indicating that the full-length APPLE microprotein is essential for these functions.

To determine whether APPLE enhances ERK1/2 phosphorylation by promoting phosphorylation or by inhibiting dephosphorylation, we reanalyzed the APPLE-specific interacting proteins (Table S3). In addition to ERK1/2, APPLE specifically interacted with phosphatase components, including the catalytic subunit of PP1 (PPP1CB), its regulatory subunit (PPP1R12A), and the regulatory subunit of PP2A (PPP2R2A), while no interaction with the upstream kinase MEK was observed (Table S3). Consistent with this, IF assays confirmed co-localization of APPLE-Flag with p-ERK1/2, PPP1CB, PPP1R12A, and PPP2R2A in HCC cells (Fig. 7G), supporting the notion that APPLE sustains ERK1/2 phosphorylation by binding to and inhibiting PP1/PP2A phosphatase activity. Together, these findings demonstrate that APPLE directly interacts with ERK1/2 and PP1/PP2A phosphatases, inhibits ERK1/2 dephosphorylation, and thereby sustains MAPK signaling to promote HCC progression.

### Transcript factor E2F1 regulates *ASH1L-AS1* expression to promote HCC progression through encoding APPLE and enhancing ERK1/2 phosphorylation

**Fig. 8:**
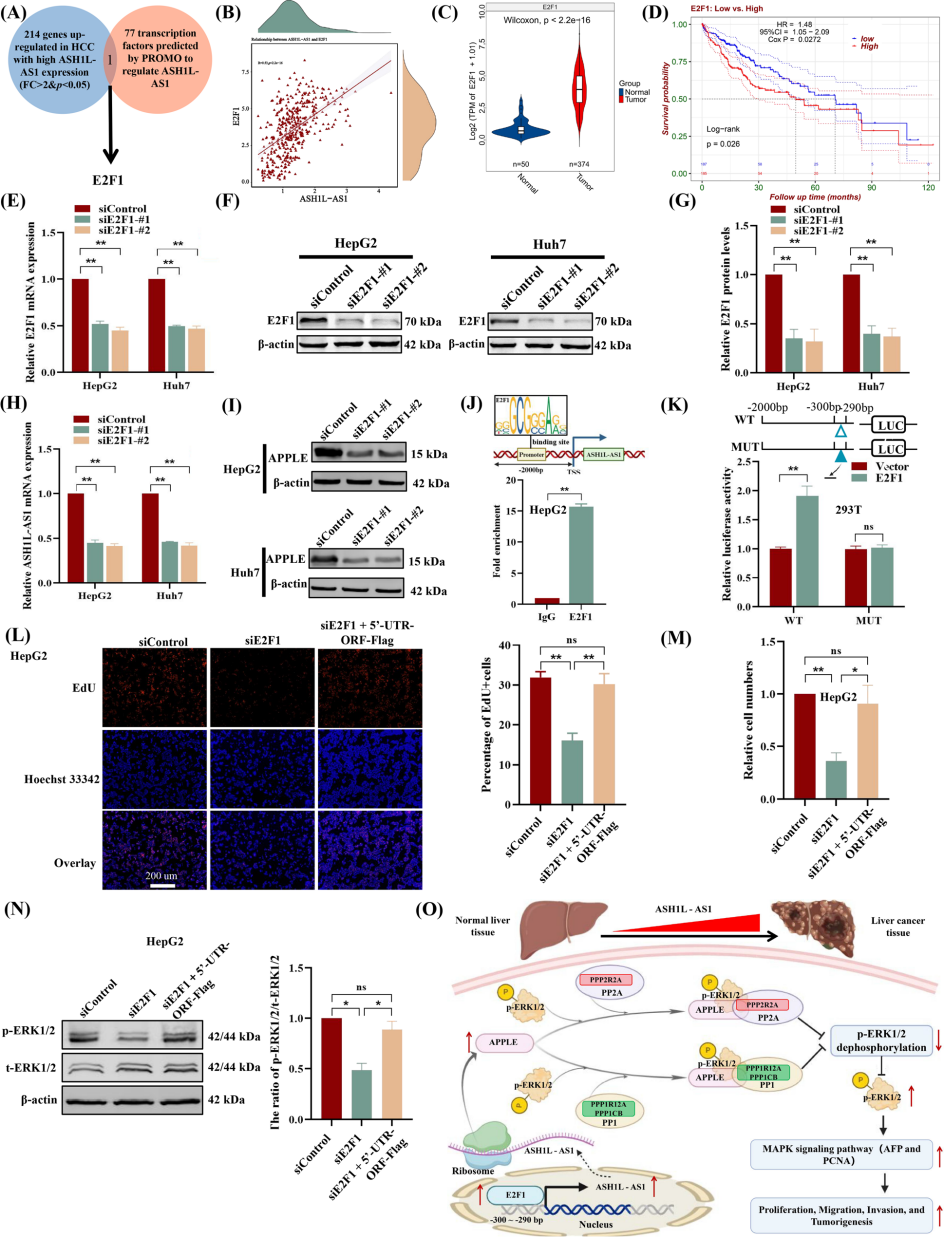
Transcription factor E2F1 binds to the *ASH1L-AS1* promoter regulating APPLE expression and enhancing ERK1/2 phosphorylation to promote HCC progression. **(A)** Transcription factor prediction using PROMO and differential expression analysis identifying E2F1 as a regulator of *ASH1L-AS1* expression. **(B-D)** TCGA-LIHC dataset analysis showing the correlation between *ASH1L-AS1* and *E2F1* expression **(B)**, differential gene expression of *E2F1* between HCC and normal tissues **(C)**, and the association of *E2F1* expression with HCC prognosis **(D)**. **(E-G)** qPCR **(E)** and Western blot (**F-G**) validation of *E2F1* knockdown efficiency. **(H-I)** qPCR and Western blot analysis confirming the effects of *E2F1* knockdown on *ASH1L-AS1* transcription (**H**) and APPLE protein expression **(I)**. **(J)** ChIP-qPCR demonstrating the specific binding of E2F1 to the *ASH1L-AS1* promoter. **(K)** Luciferase reporter assay validating the regulatory effect of E2F1 binding to the *ASH1L-AS1* promoter (-300 bp to -290 bp). **(L-N)** EdU and Transwell assays evaluating the effects of E2F1 knockdown and simultaneous APPLE rescue on HepG2 cell proliferation **(L)**, migration **(M)**, and p-ERK1/2 phosphorylation levels **(N)**. **(O)** Schematic illustration of the molecular mechanism by which the ASH1L-AS1-encoded microprotein APPLE promotes HCC progression

Given that *ASH1L-AS1* exhibits amplification or structural variation only in 6% of HCC patients (Fig. S2A), we sought to investigate other mechanisms underlying its upregulation in HCC. First, we analyzed the TCGA-LIHC dataset and identified 214 genes significantly upregulated in patients with high *ASH1L-AS1* expression (Fig. 8A and Table S4). Moreover, we used the PROMO tool [35] and predicted 77 potential transcription factors that could bind to the *ASH1L-AS1* promoter (Fig. 8A & Table S5). By intersecting the upregulated genes in HCC with the predicted transcription factors, we identified E2F1 as a potential transcriptional regulator of *ASH1L-AS1* (Fig. 8A). Further analysis confirmed that *E2F1* expression is positively correlated with *ASH1L-AS1* (Fig. 8B&C) and, like *ASH1L-AS1*, is significantly associated with the progression of HCC (Fig. 8D and Fig. S6). In addition, *E2F1* knockdown in HepG2 and Huh7 cells reduced *ASH1L-AS1* and its products APPLE expression (Fig. 8E-I). These findings suggest that E2F1 regulates *ASH1L-AS1* transcription expression.

To confirm the direct transcriptional regulation of *ASH1L-AS1* by E2F1, The ChIP assay was performed, which confirmed E2F1 binding to the *ASH1L-AS1* promoter (Fig. 8J). Consistent with the ChIP results, luciferase assays showed that E2F1 overexpression enhanced *ASH1L-AS1* promoter activity, which was abolished by mutating the potential binding site in the − 300 bp to -290 bp promoter region (Fig. 8K). Furthermore, knockdown of *E2F1* significantly reduced ERK1/2 phosphorylation and inhibited proliferation and invasion of HCC cells, while APPLE overexpression rescued these effects (Fig. 8L-N). These findings indicate that E2F1 specifically regulates *ASH1L-AS1* transcription and APPLE expression to promote HCC progression through impacting ERK1/2 dephosphorylation and activating MAPK signaling (Fig. 8O).

### Exploring therapeutic strategies targeting the E2F1-ASH1L-AS1/APPLE-ERK1/2 axis in HCC

**Fig. 9:**
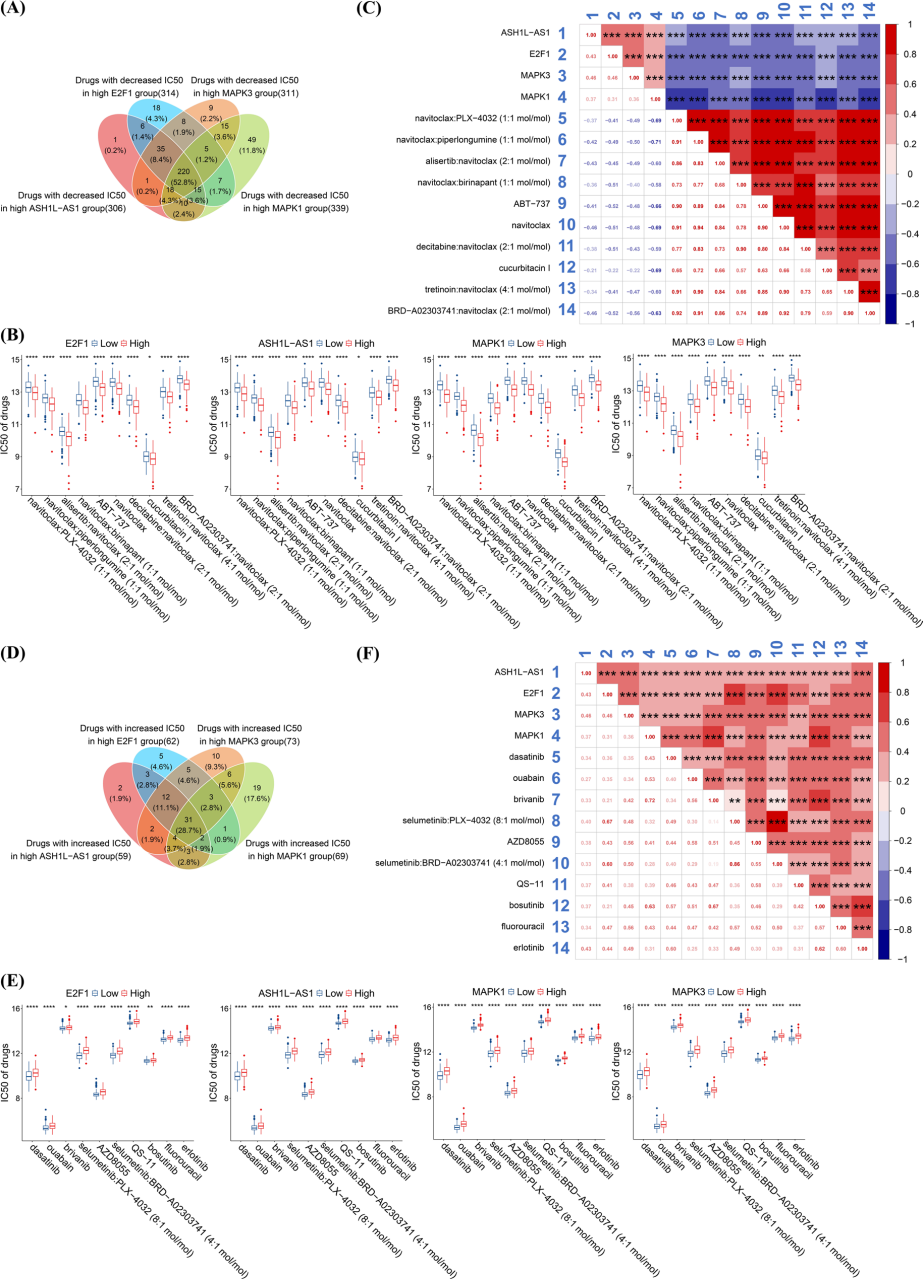
Identification of potential therapeutic agents targeting the E2F1-ASH1L-AS1-APPLE-ERK1/2 axis in HCC. **(A)** Drug sensitivity analysis based on the TCGA–LIHC dataset using OncoPredict identified 220 treatment combinations with significantly decreased IC50 values in patients with exhibiting high expression of E2F1, *ASH1L-AS1*, and ERK1/2 (*MAPK3*/*MAPK1*), suggesting potential drug sensitivity. **(B)** The top 10 most sensitive treatment combinations with the greatest IC50 reduction in the high-expression group are shown. **(C)** Expression levels of these genes were negatively correlated with IC50 values, further supporting increased drug sensitivity. **(D)** Conversely, 31 treatment combinations showed a significant increase in IC50 values in the high-expression group, indicating potential drug resistance. **(E)** The top 10 most resistant treatment combinations showed a significant increase in IC50 values in the high-expression group, indicating potential drug resistance. **(F)** In these cases, gene expression levels were positively correlated with IC50 values, indicating decreased sensitivity

To explore therapeutic strategies targeting the E2F1-ASH1L-AS1/APPLE-ERK1/2 axis in HCC, we performed drug sensitivity analysis using the TCGA-LIHC dataset, along with OncoPredict [27] and CTRP2 [28] tools. This analysis identified 220 treatment combinations with significantly reduced IC50 values in patients with high expression of *E2F1*, *ASH1L-AS1*, *MAPK1*, and *MAPK3*, indicating enhanced drug sensitivity (Fig. 9A & Table S6). The top 10 most promising treatment combinations are listed in Fig. 9B, all exhibiting a negative correlation between IC50 values and the expression levels of the axis components (Fig. 9C), suggesting these therapies may be particularly effective in high-expression patients. In contrast, 31 treatment combinations showed a significant increase in IC50 values in the same patient group, suggesting potential drug resistance (Fig. 9D & Table S6). The top 10 of these less effective combinations are presented in Fig. 9E, each showing a positive correlation between the IC50 values and the expression of *E2F1*, *ASH1L-AS1*, *MAPK1*, and *MAPK3* (Fig. 9F), implying reduced efficacy in high-expression contexts. These findings highlight the therapeutic relevance of the E2F1-ASH1L-AS1/APPLE-ERK1/2 axis in HCC and suggest the rationale for precision medicine strategies targeting this pathway. Nonetheless, further experimental validation is required to confirm these observations.

## Discussion

In this study, we systematically identified and characterized *ASH1L-AS1* as a translatable lncRNA that encodes the oncogenic microprotein APPLE, which plays a pivotal role in HCC progression. Through integrative analysis of TCGA-LIHC and TranLnc data, we identified 696 translatable lncRNAs (Fig. 1), among which *ASH1L-AS1* was prioritized based on translational evidence and clinical relevance (Table 1; Fig. 2). Functional assays confirmed that APPLE, rather than its RNA precursor, promotes ERK1/2 phosphorylation and MAPK activation, thereby driving HCC cell proliferation, migration, invasion, and tumorigenicity (Fig. 3). These effects were reversed by ERK1/2 inhibition or *ASH1L-AS1* knockdown, highlighting APPLE as a key effector of MAPK-driven oncogenesis in HCC (Figs. 4, 5 and 6).

While aberrant MAPK pathway activation is observed in over 75% of HCC cases, mutations on the classical Ras–Raf–MEK cascade are infrequent [15, 16]. Additionally, the limited efficacy of current inhibitors targeting this classical cascade in HCC [15] suggests alternative, non-canonical mechanisms may underlie MAPK activation. Our findings reveal a non-canonical mechanism by which APPLE sustains ERK1/2 phosphorylation through direct interaction with ERK1/2 and protein phosphatases PP1/PP2A, likely impairing phosphatase-mediated MAPK signaling deactivation (Fig. 7). This mechanism complements existing models of MAPK activation and offers new insights into HCC pathogenesis. Although APPLE-PP1/PP2A binding was confirmed, further studies using molecular docking and mutagenesis are required to map the specific domains mediating these interactions and to determine whether APPLE also affects upstream Ras–Raf–MEK signaling components.

Beyond its role in MAPK activation, our additional analyses suggest that ASH1L-AS1/APPLE may also contribute to shaping the tumor immune microenvironment. Specifically, high *ASH1L-AS1* expression is associated with decreased infiltration of cytotoxic CD8⁺T cells and macrophages, and enrichment of non-cytotoxic T cell subsets, indicative of an immunologically “cold” tumor phenotype (Fig. S1E). This pattern was further corroborated by immune subtype classification, which revealed elevated *ASH1L-AS1* expression in immune cold tumors (Fig. 2J). These findings suggest a potential role for APPLE in immune evasion and may explain resistance to immunotherapy in certain HCC subtypes. Moreover, *ASH1L-AS1* expression was significantly higher in female HCC patients and correlated inversely with *ESR1* but positively with *ESR2* expression, implicating estrogen receptor signaling in its sex-biased transcriptional regulation (Fig. 2F-I). Further experimental validation, such as estrogen treatment assays, will be necessary to confirm a direct regulatory role. Additionally, we observed that APPLE-mediated ERK1/2 activation is independent of RAS mutational status, highlighting its relevance as a broadly active oncogenic mechanism even in RAS wild-type tumors, which comprise the vast majority of HCC cases (Fig. S2C&D). These insights underscore the multifaceted role of ASH1L-AS1/APPLE in tumor biology, spanning proliferation, immune modulation, sex-specific regulation, and MAPK pathway reprogramming.

At the transcriptional level, our results demonstrate that transcript factor E2F1 directly binds the *ASH1L-AS1* promoter, driving APPLE overexpression in HCC (Fig. 8). Given that *ASH1L-AS1* gene amplification occurs in only ~ 6% of HCC cases, transcriptional activation appears to be the predominant regulatory mechanism. Based on drug sensitivity data, we identified treatment combinations that selectively target HCC subtypes with E2F1-ASH1L-AS1/APPLE-ERK1/2 axis hyperactivation, offering a rationale for precision therapy in this molecular context (Fig. 9). However, additional preclinical validation is needed to assess the therapeutic efficacy of these candidates. Despite the promising findings, the lack of validation in large-scale, multicenter clinical cohorts limits our ability to generalize the expression patterns, interaction networks, and prognostic significance of APPLE and its associated signaling components. This remains a key barrier to clinical translation.

From a therapeutic standpoint, although drug development targeting protein phosphatases remains challenging [36], APPLE represents an attractive target due to its critical role in sustaining oncogenic MAPK signaling. Direct inhibition strategies could involve small molecules that disrupt p–ERK1/2–APPLE–PP1/PP2A interactions, thereby restoring phosphatase activity, promoting p–ERK1/2 dephosphorylation, and suppressing MAPK hyperactivation. Alternatively, targeting upstream regulators such as E2F1 to reduce APPLE transcription offers an indirect but potentially synergistic approach. These multilayered strategies may improve therapeutic response and clinical outcomes in HCC patients.

In addition to its proliferative effects, APPLE also promotes HCC cell migration and invasion (Fig. 3D&E), indicating a potential role in metastasis—a leading cause of HCC-related mortality [37]. Consistent with this, we observed that some APPLE-specific interacting proteins are significantly enriched in cell adhesion-related pathways, including focal adhesion and cadherin binding (Fig. 7C). While our findings support the pro-metastatic potential of APPLE, it remains unclear whether this effect is mediated through classical mechanisms such as epithelial–mesenchymal transition, cell adhesion regulation, or extracellular matrix remodeling. Further elucidation of these pathways will be critical to fully understand the mechanistic basis of APPLE-driven metastasis.

Although our research focused on HCC, whether APPLE is similarly upregulated and functional in other cancer types is currently unknown. This limits our understanding of its broader oncogenic potential. Nevertheless, our findings highlight a novel paradigm in cancer biology—namely, the functional significance of microproteins encoded by lncRNAs. APPLE exemplifies how such non-canonical proteins may regulate key signaling pathways, maintain cancer stemness, and contribute to drug resistance across malignancies.

In summary, this study provides the first comprehensive characterization of APPLE as an oncogenic microprotein encoded by a lncRNA, which promotes HCC progression through a non-canonical mechanism involving sustained activation of the MAPK signaling pathway. The elucidation of the E2F1-ASH1L-AS1/APPLE-ERK1/2 signaling axis not only advances our understanding of MAPK regulation in liver cancer but also identifies a promising target for precision therapy with significant translational potential.

## Electronic supplementary material

Below is the link to the electronic supplementary material.

Additional file 1: Table S1. Details of primary antibodies for WB, IF and Co-IP. Table S2. Details of primers for qPCR. Table S3. Protein-protein interactome of the microprotein APPLE in HCC. Table S4. Differentially expressed genes between patients with high and low *ASH1L-AS1* expression. Table S5. Prediction of 77 potential transcription factors binding to the *ASH1L-AS1* promoter region using the PROMO online tool. Table S6. List of treatment combinations with higher and lower IC50 values in HCC patients with high expression of the E2F1-ASH1L-AS1/APPLE-ERK1/2 axis, indicating reduced efficacy (higher IC50) or increased sensitivity (lower IC50)

Additional file 2: Fig. S1. Associations between *ASH1L-AS1* expression and clinical features, RAS mutation status, and tumor immune microenvironment in HCC. (A–D) Violin plots illustrating the association between *ASH1L-AS1* expression and clinical characteristics in HCC patients, including tumor stage (A), clinical stage (B), age (C), and ethnicity (D). (E) Correlation plot (corrplot) showing the relationship between *ASH1L-AS1* expression and the infiltration levels of various stromal and immune cell types in the tumor microenvironment, as predicted by xCell. (F–G) Violin plots showing the association between *ASH1L-AS1* expression and metastasis status (F) and lymph node involvement (G) in HCC patients. Fig. S2. Genomic alterations and mutation landscape associated with *ASH1L-AS1* in HCC. (A) Analysis of 1,380 HCC patients from five studies in cBioPortal revealed that 6% of patients exhibited *ASH1L-AS1* amplification or structural variations. (B) Upregulated *ASH1L-AS1* is associated with higher mutation frequencies in hotspot genes in HCC. (C) Stacked bar plot showing the proportion of RAS mutations in HCC patients with high and low *ASH1L-AS1* expression. (D) Box plot showing the differential expression of *ASH1L-AS1* between HCC patients with RAS mutations and those with wild-type RAS. Fig. S3. TCGA-LIHC data analysis reveals distinct gene expression patterns and dysregulated pathways associated with *ASH1L-AS1* expression in HCC. PCA plot (A), heatmap (B), and volcano plot (C) showing significant differences in gene expression patterns between HCC patients with high and low *ASH1L-AS1* expression. (D-E) GO and KEGG enrichment analyses highlighting the functional pathways enriched in differentially expressed genes between the two groups. Correlation plots illustrating the association of enriched genes with cellular senescence (F), p53 signaling (G), and hepatocellular carcinoma-related pathways (H). Fig. S4. Overexpression of the microprotein APPLE enhances p-ERK1/2 levels in HepG2 cells. Fig. S5. Expression characteristics of APPLE-Flag-specific interacting proteins and their association with *ASH1L-AS1* in HCC. (A) Molecular weight distribution of proteins specifically interacting with the APPLE-Flag fusion protein. (B) TCGA-LIHC analysis reveals that proteins enriched by APPLE-Flag, identified via co-immunoprecipitation and proteomics, are significantly upregulated in HCC tissues with high *ASH1L-AS1* expression. (C) Expression levels of these proteins are positively correlated with ASH1L-AS1 expression across HCC samples. Fig. S6. TCGA-LIHC data analysis reveals the clinical relevance of *E2F1* gene expression in HCC. *E2F1* gene expression is significantly associated with tumor stage (A, clinical stage (B), and ethnicity (C), but shows no significant correlation with age (D) or sex (E)

## Data Availability

The transcriptomic data are stored in NCBI GEO under accession number GSE294409. Other data and study materials are available upon request from the corresponding authors.

## Abbreviations

ChIP-qPCR: Chromatin immunoprecipitation followed by quantitative PCR
Co-IP: Co-immunoprecipitation
CTRP2: Cancer Therapeutics Response Portal 2
DEGs: Differentially expressed genes
DUSP: Dual-specificity phosphatase
DMSO: Dimethyl sulfoxide
ERK1/2: Extracellular signal-regulated kinases 1 and 2
FC: Fold change
GO: Gene Ontology
GSVA: Gene Set Variation Analysis
HCC: Hepatocellular carcinoma
IF: Immunofluorescence
KEGG: Kyoto Encyclopedia of Genes and Genomes
lncRNAs: Long non-coding RNAs
MAPK: Mitogen-Activated Protein Kinase
MC: Manual curation
MS: Mass spectrometry
NS: Normal control
ORFs: Open reading frames
PCA: Principal component analysis
PCR: Polymerase chain reaction
PP1: Protein phosphatase 1
PP2A: Protein phosphatase 2 A
ShNC: shRNA control
SNV: Single nucleotide variant
WB: Western blot
