# Supplementary material for: Non-canonical activation of MAPK signaling by the lncRNA ASH1L-AS1-encoded microprotein APPLE through inhibition of PP1/PP2A-mediated ERK1/2 dephosphorylation in hepatocellular carcinoma
Source: J Exp Clin Cancer Res. 2025 Jul 11;44:200. doi: 10.1186/s13046-025-03465-w (PMC12247205; doi:10.1186/s13046-025-03465-w)
Supplement: Supplementary file 2 — Additional file 2: Fig. S1. Associations between ASH1L-AS1 expression and clinical features, RAS mutation status, and tumor immune microenvironment in HCC. (A–D) Violin plots illustrating the association between ASH1L-AS1 expression and clinical characteristics in HCC patients, including tumor stage (A), clinical stage (B), age (C), and ethnicity (D). (E) Correlation plot (corrplot) showing the relationship between ASH1L-AS1 expression and the infiltration levels of various stromal and immune cell types in the tumor microenvironment, as predicted by xCell. (F–G) Violin plots showing the association between ASH1L-AS1 expression and metastasis status (F) and lymph node involvement (G) in HCC patients. Fig. S2. Genomic alterations and mutation landscape associated with ASH1L-AS1 in HCC. (A) Analysis of 1,380 HCC patients from five studies in cBioPortal revealed that 6% of patients exhibited ASH1L-AS1 amplification or structural variations. (B) Upregulated ASH1L-AS1 is associated with higher mutation frequencies in hotspot genes in HCC. (C) Stacked bar plot showing the proportion of RAS mutations in HCC patients with high and low ASH1L-AS1 expression. (D) Box plot showing the differential expression of ASH1L-AS1 between HCC patients with RAS mutations and those with wild-type RAS. Fig. S3. TCGA-LIHC data analysis reveals distinct gene expression patterns and dysregulated pathways associated with ASH1L-AS1 expression in HCC. PCA plot (A), heatmap (B), and volcano plot (C) showing significant differences in gene expression patterns between HCC patients with high and low ASH1L-AS1 expression. (D-E) GO and KEGG enrichment analyses highlighting the functional pathways enriched in differentially expressed genes between the two groups. Correlation plots illustrating the association of enriched genes with cellular senescence (F), p53 signaling (G), and hepatocellular carcinoma-related pathways (H). Fig. S4. Overexpression of the microprotein APPLE enhances p-ERK1/2 levels in He [file 13046_2025_3465_MOESM2_ESM.pdf]

Supplementary Figures

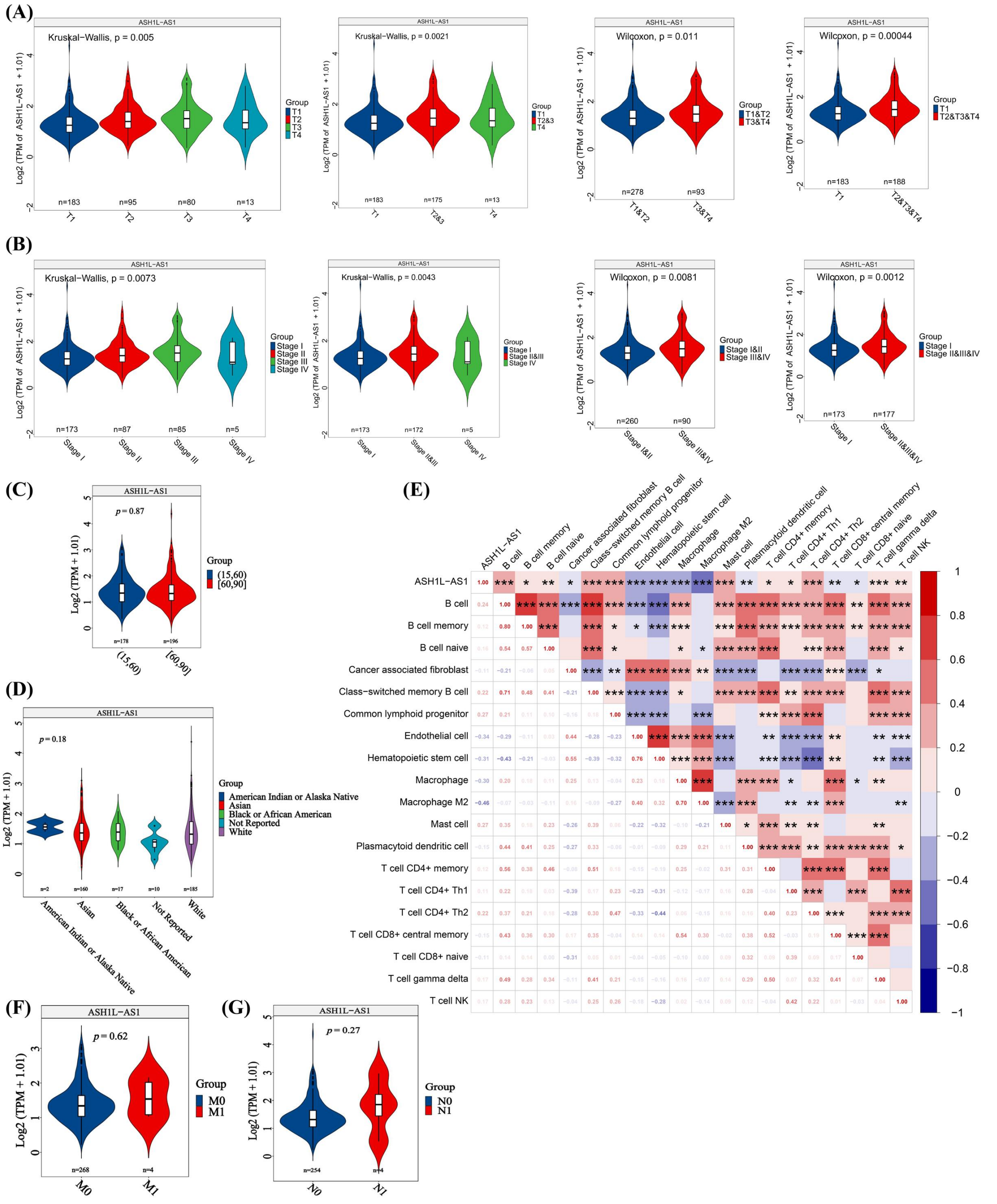

Fig. S1. Associations between *ASH1L-AS1* expression and clinical features, RAS mutation status, and tumor immune microenvironment in HCC. (A – D) Violin plots illustrating the association between *ASH1L-AS1* expression and clinical characteristics in HCC patients, including tumor stage (A), clinical stage (B), age (C), and ethnicity (D). **(E)** Correlation plot (corrplot) showing the relationship between *ASH1L-AS1* expression and the infiltration levels of various stromal and immune cell

types in the tumor microenvironment, as predicted by xCell. **(F – G)** Violin plots showing the association between *ASH1L-AS1* expression and metastasis status (F) and lymph node involvement (G) in HCC patients.

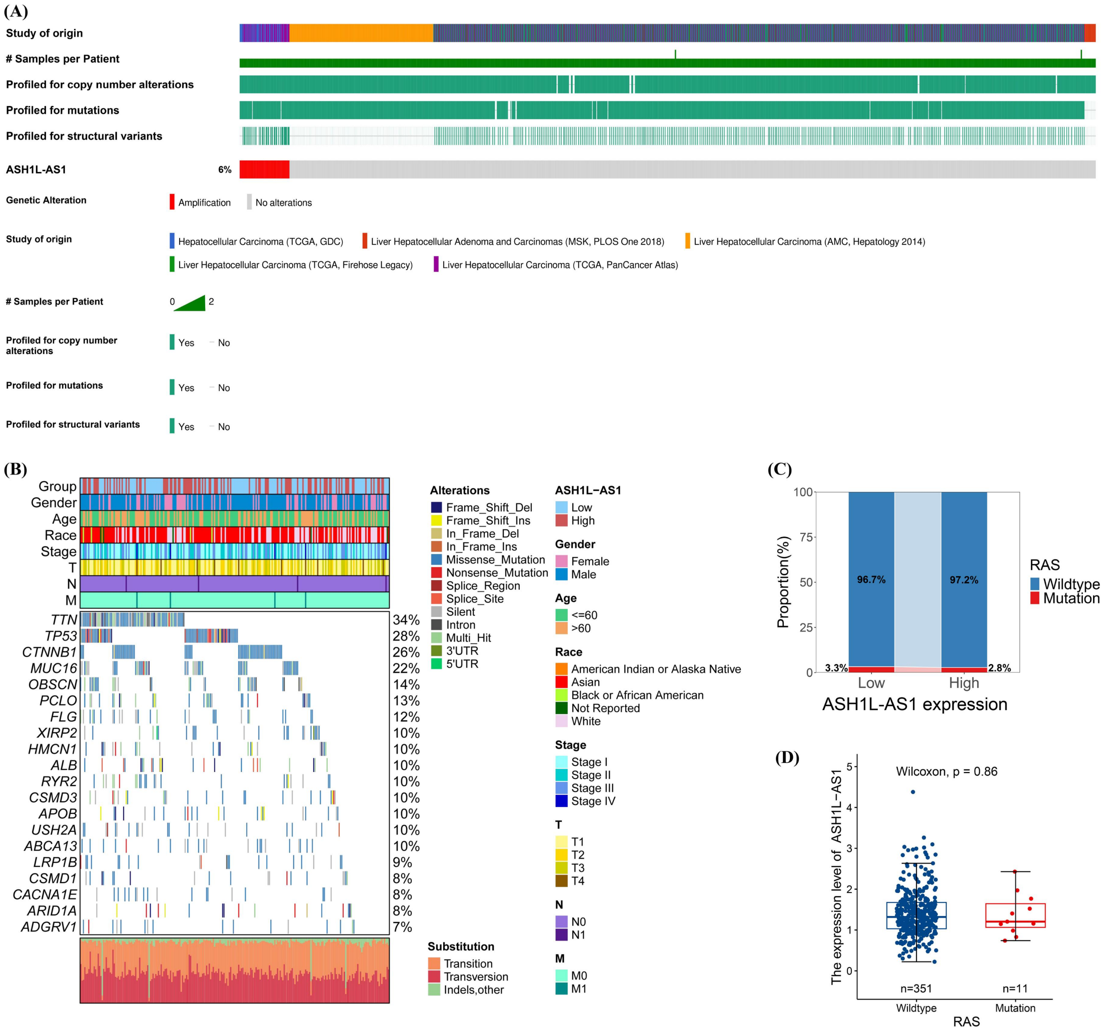

Fig. S2. Genomic alterations and mutation landscape associated with *ASH1L-AS1* in HCC. (A) Analysis of 1,380 HCC patients from five studies in cBioPortal revealed that 6% of patients exhibited *ASH1L-AS1* amplification or structural variations. **(B)** Upregulated *ASH1L-AS1* is associated with higher mutation frequencies in hotspot genes in HCC. **(C)** Stacked bar plot showing the proportion of RAS mutations in HCC patients with high and low *ASH1L-AS1* expression. **(D)** Box plot showing the differential expression of *ASH1L-AS1* between HCC patients with RAS mutations and those with wild-type RAS.

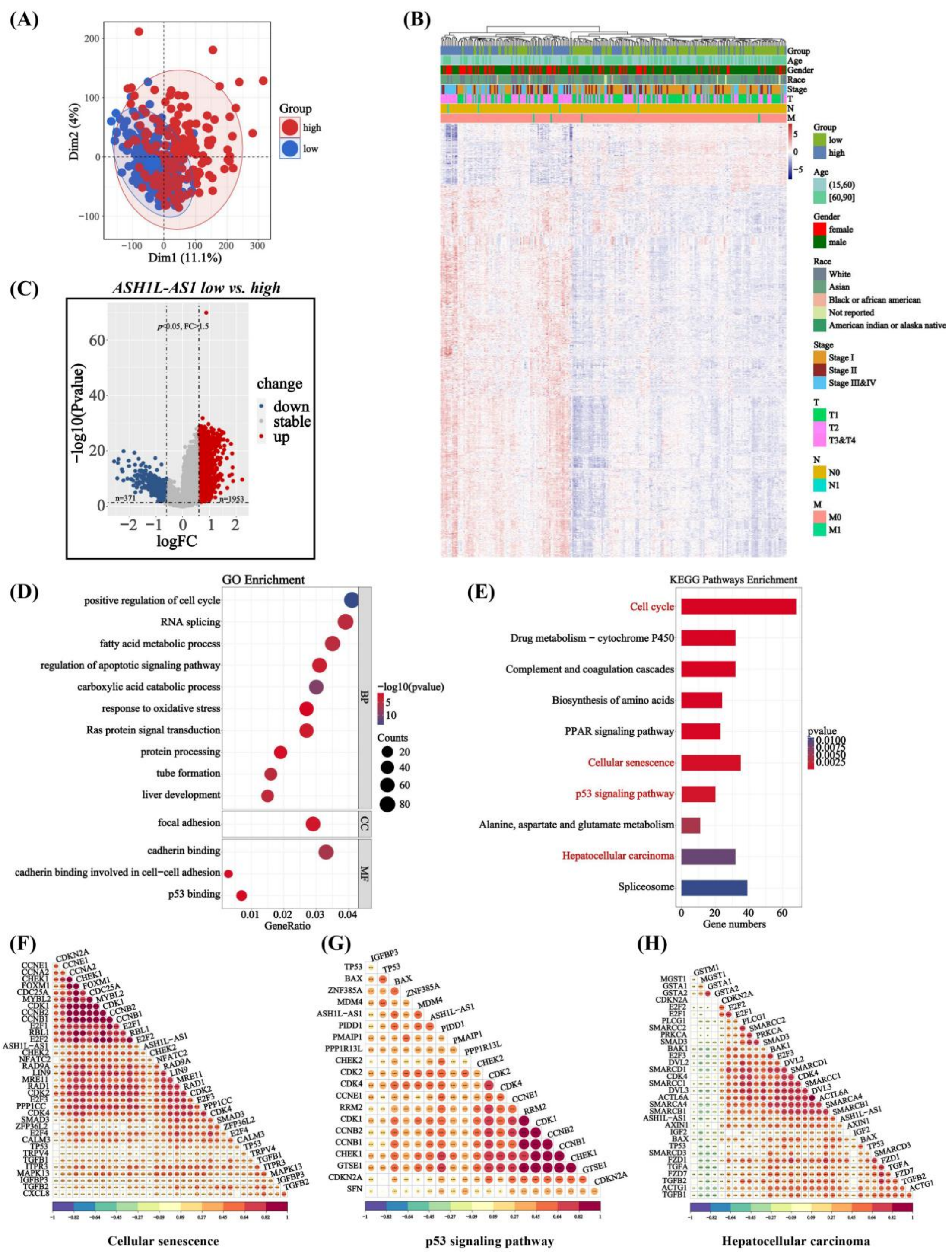

Fig. S3. TCGA-LIHC data analysis reveals distinct gene expression patterns and dysregulated pathways associated with *ASH1L-AS1* expression in HCC. (A-C) PCA plot (A), heatmap (B), and volcano plot (C) showing significant differences in gene expression patterns between HCC patients with high and low *ASH1L-AS1* expression. **(D-E)** GO and KEGG enrichment analyses highlighting the functional pathways enriched in differentially expressed genes between the two groups. **(F-H)**

Correlation plots illustrating the association of enriched genes with cellular senescence (F), p53 signaling (G), and hepatocellular carcinoma-related pathways (H).

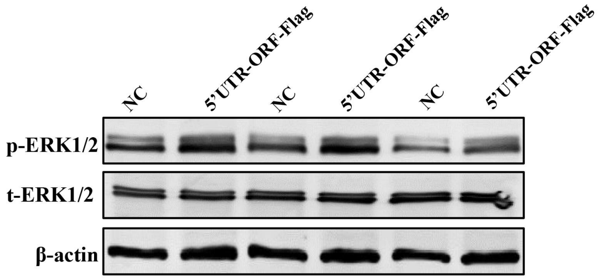

Fig. S4. Overexpression of the microprotein APPLE enhances p-ERK1/2 levels in HepG2 cells.

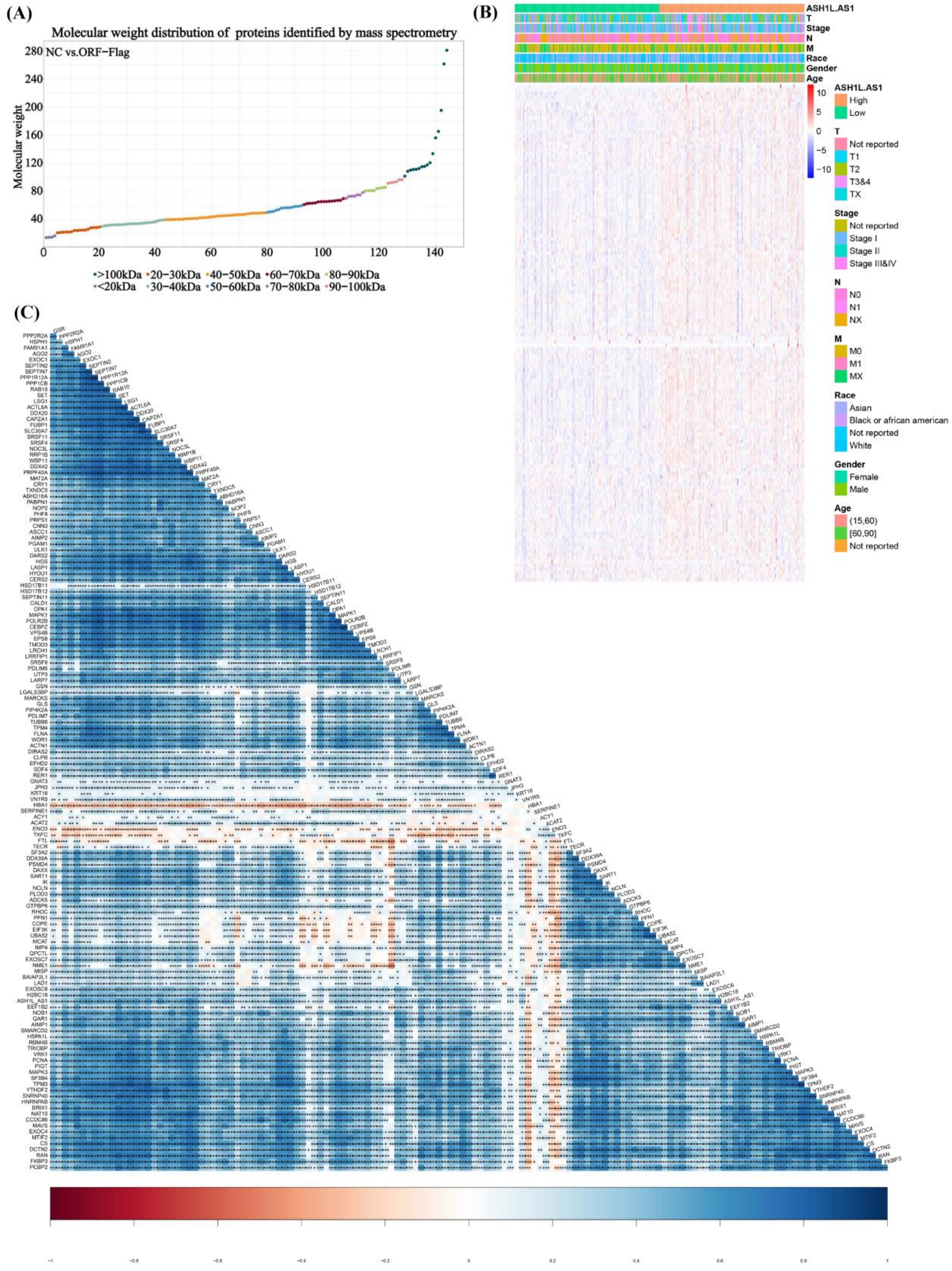

Fig. S5. Expression characteristics of APPLE-Flag-specific interacting proteins and their association with *ASH1L-AS1* in HCC. (A) Molecular weight distribution of proteins specifically interacting with the APPLE-Flag fusion protein. **(B)** TCGA-LIHC analysis reveals that proteins enriched by APPLE-Flag, identified via co-immunoprecipitation and proteomics, are significantly upregulated in HCC tissues with high *ASH1L-AS1* expression. **(C)** Expression levels of these proteins are positively correlated with *ASH1L-AS1* expression across HCC samples.

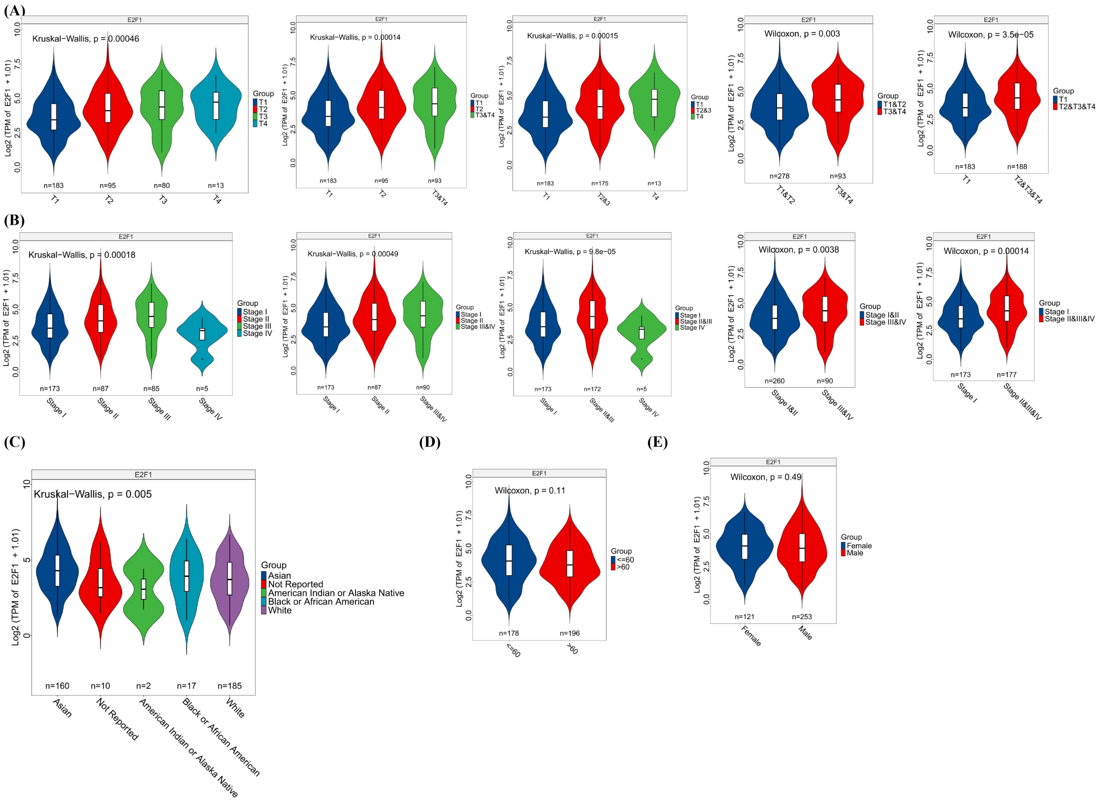

Fig. S6. TCGA-LIHC data analysis reveals the clinical relevance of *E2F1* gene expression in HCC. *E2F1* gene expression is significantly associated with tumor stage **(A)**, clinical stage **(B)**, and ethnicity **(C)**, but shows no significant correlation with age **(D)** or sex **(E)**.
